# CLL-1: An emerging target for immunotherapy in acute myeloid leukemia

**DOI:** 10.1007/s00277-026-06916-2

**Published:** 2026-03-14

**Authors:** Yu Wang, Yi Xiao

**Affiliations:** Department of Hematology, Tongji Hospital, Tongji Medical College, Huazhong University of Science and Technology, No. 1095, Jiefang Road, Qiaokou District, Wuhan, 430030 Hubei P.R. China

**Keywords:** C-type lectin-like molecule-1, Acute myeloid leukemia, Immunotherapy, Targeted therapy, Chimeric antigen receptor T cells, Antibody-drug conjugates

## Abstract

AML is an aggressive haematological malignancy characterised by uncontrolled proliferation and differentiation arrest of myeloid progenitor/stem cells. Conventional treatment methods principally entail chemotherapy and haematopoietic stem cell transplantation; however, the efficacy of these treatments is constrained by the occurrence of relapses and treatment-related toxicity. In recent years, research into molecular mechanisms has driven the development of targeted therapies against specific gene mutations and advanced multiple immunotherapy strategies. Among these, C-type lectin-like molecule 1 (CLL-1) has emerged as a promising new immunotherapy target due to its specific expression in AML blast cells and leukemia stem cells. CLL-1-targeted therapies have been shown to have the potential to alleviate drug resistance, reduce non-specific toxicity, and address issues of immune escape. This review provides a comprehensive summary of the latest research advances in CLL-1-targeted therapies for AML, with the aim of providing novel insights and directions for clinical treatment.

## Introduction

AML is one of the most common types of acute leukaemia in adults. It is characterised by high heterogeneity and aggressiveness. Its pathogenesis primarily involves the abnormal proliferation and impaired differentiation of myeloid progenitor cells [1]. Traditional treatment paradigms for AML mainly rely on chemotherapy and haematopoietic stem cell transplantation [2], however, the efficacy of chemotherapy is often limited by relapse, drug resistance and treatment-related toxicity, while transplantation is restricted by donor availability, transplant-related complications and graft-versus-host disease [3, 4]. With the rapid development of molecular pathology and genomics research, targeted drugs for specific gene abnormalities have gradually entered clinical use. Examples include the FLT3, IDH1 and IDH2 inhibitors, which have improved the prognosis of some patients [5–7]. However, due to the complex molecular spectrum and significant heterogeneity of AML, monotherapy with targeted drugs alone is insufficient to comprehensively address relapse and drug resistance. Therefore, immunotherapy for AML has recently become a popular research topic, with various strategies being investigated, including monoclonal antibodies, bispecific antibodies, and Chimeric Antigen Receptor T-cell (CAR-T) cell therapy [8–10]. Of particular interest is the CLL-1, which is highly expressed in AML blasts and leukaemia stem cells, but absent or only weakly expressed in normal haematopoietic stem cells [11–13]. This demonstrates good target specificity and safety. Research into immunotherapy centred on CLL-1 offers new ideas for overcoming the challenges of drug resistance, toxicity and immune evasion in current treatments. This article provides a systematic review of CLL-1-related research and explores its potential value in AML treatment. The aim is to promote the optimisation of precision treatment strategies and the clinical translation of novel immunotherapies.

## Biological characteristics of CLL-1

C-type lectin-like molecules (CTLs) are a large and highly diverse superfamily of proteins characterised by a carbohydrate-recognition domain (CRD) which typically relies on calcium ions (Ca²⁺) to bind specifically to certain glycan ligands [14]. These molecules are widely expressed on the surface of immune cells and play an indispensable role in biological processes such as immune recognition, cell adhesion, inflammation regulation and host defence mechanisms against pathogens [15, 16]. CLL-1, also known as CLEC12A) is an important member of this family. The CLL-1 gene is located in the p13.31-p13.2 region of human chromosome 12 and encodes 265 amino acids [11]. Structurally, CLL-1 exhibits the typical features of a type II transmembrane protein, with its extracellular segment containing a highly conserved C-type lectin-like domain [17, 18]. While CLL-1 is categorised as part of the C-type lectin family, it is important to note that its CRD domain has undergone significant amino acid substitutions, replacing the conserved ‘EPN’ motif with a ‘QPD’ motif. This results in the loss of the ability to bind to classical Ca²⁺-dependent glycans and instead confer specificity to certain endogenous ligands, such as urate crystals released during cell death [19]. This unique structural feature determines its functional orientation. At a molecular level, CLL-1 primarily transmits inhibitory signals through its intracellular segment via the immunoreceptor tyrosine-based inhibitory motif (ITIM). Upon ligand binding, CLL-1 negatively regulates cell signalling pathways mediated by activating receptors, thus acting as a ‘brake’ to maintain immune homeostasis and prevent autoimmune reactions [20]. In particular, on myeloid leukemia cells, CLL-1, as a specific surface antigen, is closely related to the survival and proliferation of tumor cells, placing CLL-1 at the core of immune surveillance. Therefore, CLL-1 holds significant value as an emerging target for AML immunotherapy. The structural differences between CLL-1 and other AML-related targets are shown in Table 1.

**Table 1:** Structural differences of common targets in AML

Target	Structural Features Signaling Pathway	Ligand-Binding Properties	Properties	Expression Pattern in AML	Expression in Healthy Tissues	Clinical Stage of Targeted Therapy
CLL-1	Single-pass transmembrane, CRD domain	Syk/Src-PI3K/AKT	Glycosylated proteins	> 90% AML blasts; high on LSCs; variable across FAB (M4/M5 high, M0/M3 low) [11, 13]	Myeloid lineage (neutrophils, monocytes, macrophages, DCs); absent on HSCs [13]	Phase I/II (CAR-T: NCT07036250; BsAb: NCT03038230; ADC: preclinical)
CD123	Three-pass transmembrane, IL-3 receptor α-chain	JAK/STAT	IL-3	80–93% AML; IL−3Rα chain; high on LSCs [21, 22]	Endothelial cells, plasmacytoid DCs, basophils; low on HSCs [21]	Phase I/II (BsAb: flotetuzumab NCT02152956; CAR-T: NCT04318678 ADC: tagraxofusp NCT02268253)
CD33	Single-pass transmembrane, Ig-like domain	ITIM-mediated phosphatase recruitment	Sialic acid derivatives	85–90% AML; expressed on LSCs [22]	Myeloid progenitors, mature myeloid cells; low on HSCs [23]	Approved (GO, 2017 reapproval); Phase I/II (CAR-T: NCT06420063; BsAb: NCT05077423)
CD47	Five-pass transmembrane, Ig superfamily ligand	Binds SIRPα, transmits “don’t eat me” signal, inhibits macrophage phagocytosis	Macrophage surface SIRPα protein	Universal expression on AML; “don’t eat me” signal [24]	Ubiquitous (all normal cells); high on RBCs, platelets [25]	Phase I/II (Magrolimab; BsAb: evorpacept)
TIM-3	Single-pass transmembrane, Ig V-like domain	No classic intracellular motif; delivers inhibitory signals via co-receptors (negative T/NK regulation)	Galactin-9, phosphatidylserine, etc.	80–90% AML; co-expressed with LSC markers; immune checkpoint [26, 27]	T cells, NK cells, monocytes (immune cells); not tumor-specific [28]	Phase I/II (Sabatolimab + HMAs); Phase III ongoing (STELLAR−001)

*AML* Acute Myeloid Leukemia, *IL* Interleukin, *DC* Dendritic Cell, *HSCs* Hematopoietic Stem Cells, *LSCs* Leukemic Stem Cells, *GO* Gemtuzumab ozogamicin, *RBCs* Red Blood Cells, *NK* Natural Killer (Cell), *HMA* Hypomethylating Agent

## Expression and clinical significance of CLL−1

### Tissue distribution and phylogenetic restriction

The expression profile of CLL-1 (CLEC12A) is characterised by significant tissue- and lineage-restriction, with its expression being predominantly confined to the hematopoietic system, particularly within myeloid cells. The CLL-1 protein has been detected on the surface of a range of terminally differentiated myeloid immune cells in peripheral blood and bone marrow. These cells include neutrophils, monocytes, macrophages, and certain subsets of dendritic cells [11]. Conversely, no substantial expression of CLL-1 has been observed in lymphoid cells, including T lymphocytes and B lymphocytes [29], highlighting its potential value as a marker of myeloid differentiation.

### Expression patterns in hematologic malignancies

The expression profile of CLL-1 is of significant clinical significance in the context of haematological malignancies. In AML, CLL-1 is persistently overexpressed on tumor cells. Research data indicate that the transmembrane form of CLL-1 glycoprotein is detectable in over 90% of AML cases [29]. Multiparameter flow cytometry analysis reveals that CLL-1 is present across all French-American-British (FAB) subtypes, albeit with divergent expression intensities that correlate with the degree of monocytic differentiation. Specifically, the highest mean positivity rates and fluorescence intensities are observed in M4 (myelomonocytic) and M5 (monocytic) subtypes, which are characterized by prominent monocytic/granulomonocytic differentiation. This elevated expression aligns with the physiological abundance of CLL-1 on normal monocytes and macrophages, suggesting that leukemic cells in these subtypes retain differentiation antigen expression patterns consistent with their monocytic lineage commitment. Conversely, M3 (acute promyelocytic leukemia, APL) demonstrates the lowest CLL-1 expression levels, likely reflecting its distinct pathogenesis driven by the PML-RARA fusion and its early myeloid progenitor origin with minimal monocytic differentiation potential. Notably, M0 (minimally differentiated AML) exhibits the weakest CLL-1 expression overall, consistent with its primitive immunophenotype characterized by limited myeloid maturation markers [11, 30]. Importantly, this subtype-specific expression pattern suggests that CLL−1 may serve not merely as a leukemia-associated antigen, but as a marker reflecting the differentiation stage and lineage fidelity of the malignant clone. Furthermore, no substantial disparities in CLL-1 expression levels have been identified among specific molecular genetic subtypes, including TP53 and NPM1 mutations [31], indicating that CLL-1 overexpression in AML is relatively independent of these common molecular alterations.

Beyond AML, CLL-1 expression demonstrates significant heterogeneity in myelodysplastic syndromes (MDS) and chronic myeloid leukemia (CML). Combined flow cytometry and RNA-sequencing analyses reveal that the positivity rate of CLL-1 on CD34 + progenitor cells in MDS patients fluctuates between 10% and 45%. In CML, CD34 + CD38- progenitor cell subsets in the chronic phase exhibit minimal to no CLL-1 expression; however, during accelerated or blast crisis phases, positivity rates surge to 25%–60%, suggesting a close correlation with leukemic transformation [18, 29]. This dynamic regulation—ranging from absent/low expression in normal early progenitors and chronic-phase CML to marked upregulation in differentiated myeloid malignancies and blast crisis—reinforces the association between CLL-1 expression and myeloid maturation. Consequently, therapeutic strategies targeting this antigen may specifically eliminate leukemia cells while minimizing impact on normal hematopoietic stem cells that lack CLL-1 expression. As research advances, therapeutic interventions targeting CLL-1 are anticipated to expand beyond AML to encompass other hematological malignancies, including high-risk MDS and blast-phase CML.

### Prognostic significance

Prognostically, CLL-1 expression levels demonstrate significant clinical correlation with patient outcomes. A study of 123 CD34 + AML patients revealed that low CLL-1 expression (CLL-1 low) was significantly associated with reduced complete remission(CR) rates (OR = 4.57) and elevated minimal residual disease(MRD) levels following induction chemotherapy [31]. Multivariate analysis confirmed that CLL-1 low status represents an independent adverse prognostic factor for both event-free survival and overall survival [31], with predictive value that transcends traditional risk stratification systems. Interestingly, this prognostic association appears somewhat paradoxical given the higher CLL−1 expression observed in more differentiated FAB subtypes (M4/M5), which are themselves associated with distinct clinical features. This suggests that within the context of CD34 + AML—a population typically enriched for less differentiated disease—retained CLL−1 expression may identify a subgroup with relatively more mature differentiation characteristics and correspondingly better treatment response. This prognostic utility aids in identifying patients with poor prognosis within intermediate and low-risk cytogenetic categories, potentially guiding therapeutic intensification strategies.

### Challenges and strategies for therapeutic translation

The specific expression of CLL-1 in myeloid tumors makes it a highly promising therapeutic target, and it has demonstrated preliminary efficacy in clinical studies [32, 33]. However, CLL-1-targeted therapy also carries risks such as bone marrow suppression and infection. simultaneously, the expression heterogeneity of CLL-1 across FAB subtypes, particularly its significantly reduced expression in M0 (differentiated) and M3 (acute promyelocytic leukemia) subtypes, poses substantial challenges to monotherapy strategies. Single-antigen targeting may prove insufficient for complete eradication of malignant clones. Therefore, to further enhance the efficacy of CLL-1-targeted therapies and overcome antigen escape induced by single-target approaches, multi-target combination strategies are emerging as a key direction for preventing antigen escape-mediated relapse. Currently, bispecific antibodies targeting CLL-1 (e.g., CLL-1/CD3 BsAbs) and composite CAR-T cells (e.g., CLL-1/CD33 cCAR-T) have both demonstrated encouraging clinical efficacy [34, 35]. Additionally, combination therapies like CLL-1/CD123 tandem CAR-T cells have demonstrated enhanced cytotoxicity against CD123+/CLL-1 + leukemia cells [36]. These multi-target strategies effectively address treatment challenges posed by FAB classification heterogeneity by simultaneously targeting non-overlapping antigen expression profiles, providing a crucial pathway to overcome resistance mechanisms mediated by single-antigen loss mutations.

## CLL-1-Mediated signaling pathways

CLL-1, a member of the C-type lectin-like receptor family, contains an immunoreceptor tyrosine-based inhibitory motif (ITIM) in its cytoplasmic tail. It can thus be concluded that the core characteristic of the signal transduction mechanism under investigation is the transmission of inhibitory signals as opposed to activating signals [11]. Upon binding of CLL-1 to its ligands, such as sodium urate crystals or as-yet-unidentified cell surface molecules, Src family kinases are rapidly phosphorylated and recruit SHP-1/2 phosphatases, forming a myeloid-specific negative regulatory node. This node dephosphorylates neighbouring Syk, TRAF6, or IKKβ, thereby interrupting the TLR4-NF-κB and FcγR-ROS cascades [29]. The resultant effect of this process is a decrease in IL-12p70, an increase in IL-10, and the inhibition of neutrophil calcium flux and superoxide release. In summary, in AML, the binding of CLL-1 to its ligands weakens the innate immune clearance of leukemia cells by the body. Concurrently, the downregulation of IL-12 and TNF-α, concomitant with the upregulation of IL-10, further induces the bone marrow microenvironment into an immunosuppressive state, thereby engendering conditions conducive to chemotherapy resistance.

## CLL-1 and immune cells

**Fig. 1:**
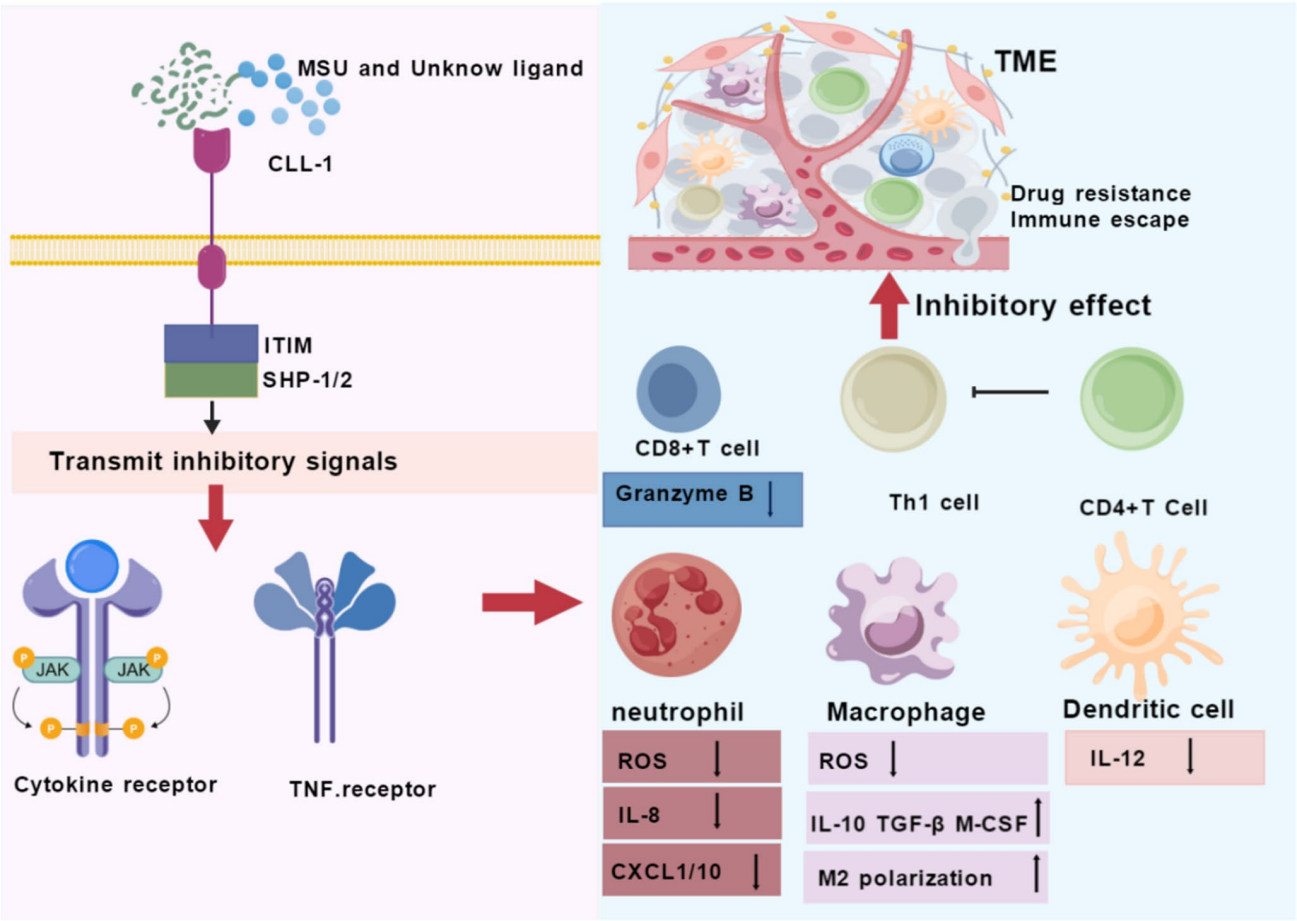
CLL−1-mediated immunoinhibitory signaling in the AML microenvironment. Binding of MSU or unknown ligands to CLL-1 recruits SHP-1/2 via the ITIM motif, inhibiting downstream cytokine and TNF receptor signaling. In the TME, this suppresses neutrophil oxidative burst and chemotaxis, promotes macrophage M2 polarization, reduces dendritic cell IL-12 production, and impairs Th1 differentiation and CD8 + T cell cytotoxicity, driving immune escape and drug resistance

Upon binding to its ligands, CLL-1 transmits inhibitory signals to multiple immune cells via the ITIM-SHP-1/2 axis [37], as demonstrated in Fig. 1. In the case of neutrophils, this pathway has been shown to rapidly block the binding of the NADPH oxidase complex within minutes, thereby inhibiting the production of superoxide anions (ROS) and IL-8, and downregulating the expression of chemokines such as CXC chemokine ligand 1 (CXCL1) and CXC chemokine ligand 10 (CXCL10). This results in the limitation of the recruitment and infiltration of neutrophils at sites of inflammation, whilst concurrently reducing calcium flux and degranulation, thus giving rise to a hyporesponsive or “quiescent” phenotype [11, 38]. In monocytes and macrophages, SHP-1/2-mediated dephosphorylation similarly inhibits NOX2 activity, leading to reduced levels of extracellular and mitochondrial ROS [39, 40]. Reactive oxygen species (ROS) are pivotal molecules for macrophages in terms of executing oxidative damage and immune effects. It has been demonstrated through a range of studies that when the levels of ROS produced by macrophages are reduced by antioxidants or chenodeoxycholic acid (CDCA), the lipid peroxidation signal in the co-culture system with AML is also reduced. This has the effect of decreasing the apoptosis rate of the leukaemia cells and increasing their proliferation [41], The mechanism of this process is thought to lie in the disruption of the ROS-p38 MAPK-DGAT1 axis, reduced lipid droplet accumulation, and blocked ferroptosis, leading to decreased sensitivity to chemotherapy or targeted drugs. Furthermore, a low-ROS environment has been shown to drive macrophages to polarise towards the M2 type, accompanied by increased secretion of IL-10, TGF-β, and M-CSF [42], which in turn inhibits Th1 responses and promotes the self-renewal of leukemia stem cells [43, 44]. This inhibitory signal has been demonstrated to downregulate the levels of CD80/CD86 in dendritic cells and to reduce IL-12 secretion. Consequently, it has been shown to inhibit the polarization of CD4 + T cells towards Th1 and to downregulate the expression of granzyme B in CD8 + T cells. This, in turn, has been shown to weaken the cytotoxic T lymphocyte (CTL) response [29]. Consequently, despite the absence of CLL-1 expression on the surface of NK/T cells, it is capable of exerting an indirect inhibitory effect on the adaptive immune effector functions by means of altering the cytokine network.

## CLL-1 endocytosis kinetics

The endocytic kinetics of CLL-1 are pivotal in determining its potential for development in antibody-drug conjugate (ADC) and CAR-T cell therapies. Research has demonstrated that the cytoplasmic tail of CLL-1 contains a canonical endocytic signal, thereby enabling rapid internalisation via clathrin-mediated endocytosis (CME). In experimental conditions, significant intracellular vesicle localization can be observed within a timespan of 15–30 min. Fluorescence pulse-chase experiments indicate that over 70% of CLL-1 molecules enter early endosomes within one hour, followed by sorting to late endosomes/lysosomes, with a degradation rate higher than the recycling rate [45]. This characteristic is advantageous for ADCs, as the antibody-bound drug can rapidly internalize and accumulate in lysosomes, efficiently releasing the payload. Preclinical models have confirmed that CLL-1-ADCs (with PBD or DM4) achieve an IC50 of less than 50 pmol·L⁻¹ against primary AML cells, with no target mutations observed in resistant strains [46]. This finding indicates that the antigen does not evade elimination through endocytic depletion.

In the context of CAR-T therapies, the endocytic kinetics have been demonstrated to exert a significant influence on the signal persistence and exhaustion profiles. The rapid internalisation of CLL-1 has been demonstrated to significantly reduce target antigen levels in the immunological synapse within a short period, which in turn leads to the indirect attenuation of the first signal. Whilst this process assists in the reduction of tonic stimulation and the delay of T cell exhaustion, excessive downregulation has also been demonstrated to induce instability within the synapse and to diminish continuous killing. Experiments have demonstrated that when the binding affinity of the CAR domain is reduced to 1–5 µmol·L-1 and a 4-1BB co-stimulatory domain is employed, phosphorylation signals can be sustained post-endocytosis, thereby achieving a balance between the efficacy of killing and the longevity of T cells [47]. It is important to note that high levels of reactive oxygen species (ROS) in the AML microenvironment have been shown to inhibit endosome acidification [48], thereby slowing the delivery of CLL-1 to lysosomes. This results in decreased ADC payload release efficiency and prolonged CAR signalling. Concurrent administration of NADPH oxidase inhibitors has been demonstrated to restore lysosomal acidity and reverse resistance. In summary, the “moderately rapid, degradation-dominant” endocytic kinetics of CLL-1 provide ADCs with the advantage of high lysosomal payload accumulation and create a low tonic stimulation environment for CAR-T therapies.

## CLL-1 targeted therapy strategies

**Fig. 2:**
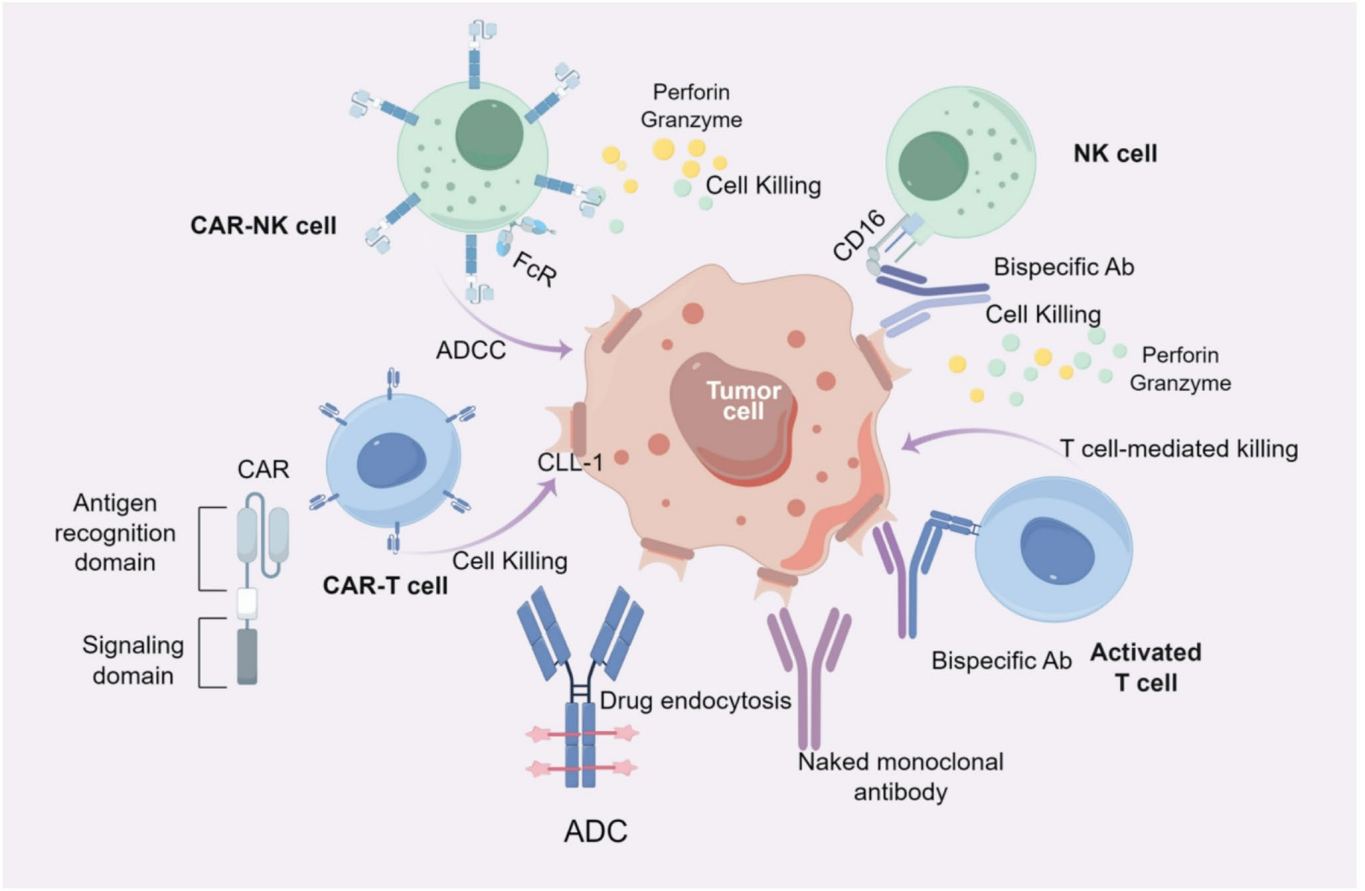
Schematic overview of CLL-1-directed immunotherapy modalities: naked monoclonal antibodies, internalizing ADCs, bispecific immune cell engagers, and adoptive CAR-T/NK cellular therapies

In light of the distribution characteristics and specificity of CLL-1 in AML, researchers are exploring novel immunotherapies targeting CLL-1. These include naked monoclonal antibodies, CLL-1-directed CAR-T cells, CLL-1 CAR-NK cell therapy, as well as ADCs, bispecific antibodies and trispecific antibodies based on the CLL-1 target (see Fig. 2).

### Naked monoclonal antibody

In consideration of the distribution characteristics and specificity of CLL-1 in AML, researchers have sought to explore the potential application of CLL-1 monoclonal antibodies in the treatment of AML. In preclinical experiments, it has been confirmed that unconjugated anti-CLL-1 antibodies relying solely on natural immune effects can exert strong anti-leukemia effects [49]. The murine anti-CLL-1 naked monoclonal antibody (clone 14C2) has been demonstrated to induce complement-mediated toxicity in 16 primary AML samples of various genetic subtypes, with 94% of the samples exhibiting dose-dependent complement activation and lysis. Furthermore, when peripheral blood mononuclear cells were employed as effector cells, the average antibody-dependent cellular toxicity (ADCC) activity exceeded 60%. In xenograft mouse models, this antibody treatment regimen has been shown to reduce the human CD45 + cell burden in the bone marrow and to extend the median survival of tumor-bearing mice from 17 to 28 days [45]. Although CLL-1 monoclonal antibodies have demonstrated ADCC/CDC activity in preclinical studies, their safety and efficacy have yet to be substantiated in human experimentation. In comparison with anti-CD33 antibodies, the antibody in question, despite being internalised by half within a period of half an hour and preferentially delivered to lysosomes, did not demonstrate a reduction in its effector function. Conversely, the accelerated antigen clearance diminished membrane epitope evasion, thereby establishing a theoretical framework for the subsequent clinical progression of antibody-drug conjugates (ADCs).

### Antibody-drug conjugate (ADC)

#### ADC drug

CLL-1 is almost exclusively confined to the surface of leukemia stem cells and is negative on hematopoietic stem cells, providing an ideal target for ADCs, which can significantly reduce off-target toxicity [50]. Furthermore, the rapid internalisation of the latter provides a natural foundation for the delivery of highly active cytotoxic drugs. It is evident that research on monoclonal antibodies targeting CLL-1 is currently accelerating towards the development of ADCs. This has become a significant strategy for the therapeutic development of this target. To date, two CLL-1 ADCs targeting AML have been systematically evaluated:

The first compound is DCLL9718S, represents a first-generation ADC approach that encountered critical clinical limitations. It is a THIOMAB™ antibody-drug conjugate (TDC) consisting of a humanized monoclonal IgG1 anti-CLL-1 antibody (MCLL0517A) linked to two pyrrolobenzodiazepine (PBD) dimer drugs via a cleavable disulfide linker [51]. The PBD dimers function as DNA alkylating and cross-linking agents, forming covalent bonds with DNA in the minor groove upon release [11]. A Phase I dose-escalation trial in 18 adult patients with relapsed/refractory AML was prematurely terminated due to a lack of efficacy and significant toxicity [33]. The results demonstrated an objective response rate of zero, and as many as 67% of patients experienced treatment-related adverse events of grade 3 or higher. Notably, dose-limiting toxicity manifested as hepatic injury, a class effect observed with PBD-based payloads. The failure of DCLL9718S exemplifies the limitations of first-generation ADCs: suboptimal linker stability, heterogeneous drug-to-antibody ratio (DAR), and the intrinsic immunogenicity and rapid clearance associated with early conjugation technologies [52].

In contrast, CLT030 represents a next-generation ADC with optimized molecular architecture. Its composition includes a humanised IgG1 framework conjugated to the auristatin class microtubule inhibitor DUBA (a dolastatin-10 derivative) via a cleavable valine-citrulline linker with a PEG-8 spacer. This design incorporates two engineered cysteine residues for site-specific conjugation, achieving a homogeneous DAR of 1.7–1.8 with > 95% monomer purity. The valine-citrulline linker demonstrates superior plasma stability compared to the disulfide linker in DCLL9718S, as evidenced by maintained total antibody and ADC levels in human plasma for up to 5 days. Preliminary clinical studies indicate significant anti-leukemic properties: in vitro, CLT030 demonstrated picomolar IC₅₀ (0.5–27 ng/mL) against various AML cell lines (MOLM-13, OCI-AML2, HL-60), with killing efficiency positively correlated with target antigen abundance (R² = 0.87). At the primary cell level, treatment of 14 AML bone marrow samples resulted in > 80% reduction in leukemia colony-forming ability with limited impact on normal hematopoietic stem cells. In a primary xenograft model, a single intravenous dose of 0.3 mg/kg reduced peripheral blood human CD45 + cells by approximately 22-fold within 4 weeks, with bone marrow leukemia stem cells reduced below detection limits [46]. No significant weight loss or hematological toxicity was observed.

While CLT030 has demonstrated promising preclinical efficacy in AML models, its clinical development status remains unclear; no Phase I trial results have been published to date. This stands in contrast to DCLL9718S, which completed Phase I evaluation albeit with disappointing results. The lack of clinical progression for CLT030 may reflect the challenges faced by biotechnology companies in advancing ADC therapeutics, or strategic reprioritization following the demonstration of clinical proof-of-concept by other CLL-1-targeted modalities such as MCLA-117.

#### Optimization of ADC drugs

As a crucial strategy for achieving targeted therapy against CLL-1, the development of ADCs still faces multiple challenges. To reverse the termination of DCLL9718S due to high toxicity, subsequent optimization strategies can achieve breakthroughs in three core technologies: Drug to Antibody Ratio (DAR), linker chemistry, and payload selection.

Antibody Affinity and Specificity: First-generation ADCs like DCLL9718S employed conventional humanized antibodies without fine-tuned affinity modulation. Emerging evidence suggests that ultra-high-affinity antibodies may promote rapid internalization and lysosomal degradation, thereby narrowing the therapeutic window. Next-generation technologies employ site-specific conjugation techniques—including engineered cystine disulfide bonds, non-natural amino acid click chemistry, enzyme-catalyzed transamidation, and disulfide bond reconfiguration—to generate homogeneous ADCs with defined drug-to-antibody ratios (typically DAR 2) [53–55]. These approaches enhance ADC stability, reduce toxicity to normal myeloid cells (particularly CLL-1-expressing neutrophils), and enable higher dose administration.

Linker Stability and Hydrophilicity: The disulfide-linked linker in DCLL9718S exhibited insufficient plasma stability, leading to premature payload release and off-target toxicity [56, 57]. In contrast, CLT-030 employs a valine-cystine protease-cleavable linker and enhances hydrophilicity by introducing a PEG-8 spacer, thereby improving solubility and pharmacokinetic properties [46]. Third-generation ADCs further explore sulfonate self-destruct linkers and β-glucuronidase-cleavable linkers to enhance tumor selectivity [58].

Payload potency versus bystander effects: While the PBD dimer in DCLL9718S demonstrated potent efficacy, it exhibited significant hepatotoxicity—a limitation absent in the auristatin-based DUBA payload of CLT030 [46]. However, AML exhibits substantial antigenic heterogeneity, with refractory leukemic stem cells often displaying low or absent CLL-1 expression [59]. While CLL-1-targeted ADCs effectively eliminate CLL-1 positive cells, CLL-1 negative subpopulations may survive and drive relapse. To address this, next-generation ADCs employ membrane-permeable payloads (e.g., topoisomerase I inhibitor SN-38) to eliminate neighboring low-expressing or negative tumor cells via bystander killing effects [60]. This strategy directly counters antigenic heterogeneity challenges posed by CLL-1 expression variations across FAB subtypes, particularly in low-CLL-1-expressing M0 and M3 subtypes.

This technological evolution—from random conjugation using unstable linkers to site-specific, hydrophilic, stable linker-payload systems—reflects the overall advancement of ADC technology. First-generation ADCs exhibited issues such as high immunogenicity, unstable linkers, and inconsistent DAR values. While second-generation ADCs improved payload potency and linker stability, they retained random conjugation characteristics. Third/fourth-generation ADCs like CLT-030 integrate site-specific conjugation, optimized DAR, and hydrophilic linkers, representing the current frontier in CLL-1 targeted therapy. Future directions include developing dual-payload ADCs combining different mechanisms of action, such as microtubule inhibitors and DNA-damaging agents, to overcome resistance and prevent relapse caused by antigen escape [61].

### Bispecific antibodies (BsAb)

#### Classification and mechanisms of bispecific antibodies

Bispecific antibodies (BsAbs) are a class of engineered antibodies that possess the ability to bind to two distinct antigens or epitopes in a simultaneous manner. The design of BsAbs overcomes the limitation of traditional monoclonal antibodies (mAbs) that target only a single antigen, achieving more complex therapeutic mechanisms through synergistic binding effects [62, 63], BsAbs have shown great potential in the treatment of hematological malignancies. Based on their mechanisms of action, BsAbs can be categorized into three major types: cell-bridging, dual-target inhibition, and immune modulation [64]. Cell-bridging BsAbs principally utilise one binding arm to recognise antigens on tumor cell surfaces, such as CLL-1, CD33, and CD123 on AML cells, whilst the other arm recognises activating receptors on the surface of immune effector cells. The formation of an immunological synapse between tumor and immune cells has been demonstrated to facilitate the specific elimination of tumor cells by immune cells [65–67], A notable example of this phenomenon is the utilisation of Blinatumomab, a drug that is extensively employed in the treatment of acute lymphoblastic leukaemia (ALL) [68]. Furthermore, these antibodies have been observed to induce the secretion of pro-inflammatory cytokines, such as interferon-γ and tumor necrosis factor-α. These, in turn, have been shown to inhibit the proliferation of leukaemia cells, activate macrophages and dendritic cells in a coordinated manner, and reshape the bone marrow microenvironment [69]. Furthermore, interferon-γ has been shown to upregulate MHC-I expression on tumor target cells, thereby indirectly enhancing adaptive immune recognition and further exerting anti-tumor effects [70], The concept of dual-target inhibition BsAbs refers to antibodies that simultaneously bind to two different signalling molecules or receptors on the same cell, thereby blocking two independent signalling pathways at the same time. This is believed to prevent compensatory activation that may occur subsequent to single-target inhibition. By retaining Fc-mediated ADCC/ADCP, these antibodies enhance the cytotoxic effects [71]. Examples of such dual receptor inhibition include that of EGFR × IGF-1R, PDGFRα × PDGFRβ, and receptor-ligand dual inhibition of VEGF × DLL4 [72, 73].The focus of immune modulation BsAbs is twofold, targeting both tumor-associated antigens (TAAs) and immune checkpoint molecules, including PD-1/PD-L1 and CTLA-4 [74]. Despite their present lesser application in the context of hematological malignancies, these agents have demonstrated significant efficacy in the treatment of solid tumors. The mechanism of action of these agents involves the concentration of the drug in the tumor microenvironment(TME) through the TAA arm, thereby effectively reducing systemic toxicity caused by peripheral immune activation. Furthermore, by obstructing inhibitory signals via the checkpoint arm, they directly alleviate T-cell functional suppression. This dual action engenders a high-concentration immune-stimulating environment, locally activating CD8 + T cells and depleting immunosuppressive regulatory T cells, thereby reshaping the tumor immune microenvironment and enhancing anti-tumor immune killing [75]. Examples of this include PD-L1 × CD3, PD-L1 × CTLA-4 bispecific antibodies [76–78].

#### Bispecific antibodies targeting CLL-1

The development strategy for CLL-1-targeted BsAbs is currently focused on cell-bridging BsAbs with a CLL-1/CD3 dual-target design. MCLA-117, a pioneering bispecific antibody, has been developed to target CD3 on T cells and CLL-1 on leukemia cells. This results in the redirection and activation of T-cell subsets in a manner that is independent of MHC or costimulatory signals. Van Loo et al. demonstrated that 10 ng/mL of MCLA-117 can induce > 90% lysis of primary AML blasts, with an EC50 range of 0.5–2.5 ng/mL, significantly lower than that of the CD33×CD3 control antibody (EC50 20–50 ng/mL) [79]. In mouse models, a single intravenous injection of 0.5 mg/kg MCLA-117 reduced the level of human CD45 + cells in the bone marrow to the detection limit (< 0.1%) within 7 days, while the control group remained between 60 and 80%; no grade 2 or higher cytokine release syndrome(CRS) or significant weight loss was observed. Subsequent toxicology studies conducted on cynomolgus monkeys demonstrated that, even at the maximum dose of 10 mg/kg, there was no persistent T-cell depletion or substantial organ pathology, thereby establishing a safety foundation for the drug to enter first-in-human clinical trials [34]. Furthermore, ABL602 is a cell-bridging BsAb with an asymmetric 2 + 1 architecture, comprising two CLL-1 Fabs and one CD3ε Fab. This results in a reduction of CD3 affinity to approximately 60nM. The weak CD3 binding forces the antibody to first form a high-concentration platform on tumor cells via bivalent CLL-1, followed by local accumulation of multiple weak signals to activate T cells, thereby avoiding excessive peripheral activation. In vitro experiments demonstrated that ABL602 has an EC50 of 0.05–0.3 ng/mL for primary AML, which is comparable to that of a potent control antibody. However, it exhibited a 60–80% reduction in the peak levels of IFN-γ, IL-6, and TNF-α (*P* < 0.01). In an NSG mouse model with primary AML-PBMCs, a dose of 0.02 mg/kg (administered twice weekly) reduced human CD45 + cells in the bone marrow by more than two logs. The ABL602 group exhibited stable body weight, and no grade > 2 toxicity was observed. In cynomolgus monkeys, a dose of 10 mg/kg resulted in only transient T-cell depletion (≤ 30%, recovering within 48 h) without persistent organ damage, supporting its advancement to clinical trials [80], These findings demonstrate the therapeutic potential of CLL-1 -targeted BsAbs in the field of AML. The current research results for CLL-1 targeted BsAbs are summarised in Table 2.

#### Limitations and optimization strategies for CLL−1 -targeted BsAbs

Despite the considerable promise of bispecific antibodies (BsAbs) targeting CLL-1 for the treatment of AML, there are several significant challenges and limitations to be addressed. The most critical challenge is that of target-related toxicity. Despite the absence of CLL-1 expression in hematopoietic stem cells, its presence has been documented on normal myeloid progenitor cells, monocytes, and granulocytes. Consequently, treatment invariably results in myeloid suppression [12]. Tumor heterogeneity and antigen escape have been identified as significant factors contributing to the failure of BsAb therapy [81]. Additionally, the inhibitory cytokines secreted by regulatory T cells and myeloid-derived suppressor cells have the capacity to weaken the function of effector cells, thereby limiting the efficacy of BsAbs [67]. Despite the fact that CLL-1 targeted BsAbs (for example, ABL602) have reduced CD3 affinity in order to mitigate CRS, CRS remains a significant risk that must be continuously balanced in T-cell-redirecting therapies.

In order to address these challenges, the field is actively exploring various strategies to enhance efficacy and overcome these bottlenecks. In order to address treatment-related myeloid suppression, there is a necessity to establish standardised supportive care protocols. Such protocols should include the prophylactic use of granulocyte colony-stimulating factors and other supportive therapies for active management. In order to enhance efficacy, a combination of BsAbs with hypomethylating agents has been demonstrated to alter the tumor epigenetic state, increase target antigen density, and improve the microenvironment [82, 83]. Furthermore, the construction of dual-target molecules that target both CLL-1 and other antigens can effectively prevent relapse due to the loss of a single antigen. In molecular design, the optimisation of pharmacokinetic properties and activation strength through Fc segment genetic engineering is intended to achieve an enhanced therapeutic window [84], Consequently, a comprehensive investigation of the aforementioned strategies will furnish a substantial theoretical foundation for overcoming the application limitations of BsAbs.

**Table 2:** Bispecific Antibody (BsAb) Studies Targeting CLL-1

Drug	Targets	Format	In Vitro Efficacy (EC50)	Key Translational Efficacy	Study Stage	First-in-Human Trial
CLL1-TriKE [85]	CD16a×IL-15 × CLL-1	TriKE(NK engager)	Not disclosed	Induces NK-cell killing of primary AML/LSCs at 0.5–5 ng/mL; significantly reduces leukemic burden in NSG mouse BM with ≤Grade 2 toxicity.	Preclinical	Not yet initiated
MCLA-117 [34]	CLL-1 ×CD3	2:1 IgG4 BsAb	68 ± 37 ng/mL (HL60)	> 99% leukemia clearance; targets primary AML cells and LSCs	Phase I	NCT03038230
ABL602 [80]	CLL-1 × CD3	T-cell engager (2:1)	0.05–0.3 ng/mL	> 2-log leukemia burden reduction at low dose (0.02 mg/kg)	Preclinical	Not yet initiated
CLEC12A-ENG / CLEC12A-ENG.CD123IL7Rα (DART) [86]	CLL-1×CD3 + CD123×IL-7Rα	DART-ENG (dual)	0.8 ng/mL	> 95% leukemia clearance; dual-targeting strategy reduces relapse risk	Preclinical	Not yet initiated
QL325	CLEC12A×CD3	2 + 1 valent BsAb	Not disclosed	Dose-dependent AML suppression in vivo	Preclinical	Not yet initiated
CD3 × CLL-1 [87]	CD3×CLL-1	2:1 IgG4-like BsAb	Not disclosed	Complete tumor elimination with single dose (1 mg/kg)	Preclinical	Not yet initiated

*BsAb* bispecific antibody, *AML* Acute Myeloid Leukemia, *NK* Natural Killer (cell), *LSC* Leukemia Stem Cell, *BM* Bone Marrow, *DART* Dual Affinity Re-targeting Technology, *ENG* Engineered

### Trispecific antibodies (TsAbs)

Trispecific antibodies (TsAbs) are engineered to bind to three different antigens, typically including two tumor-associated antigens (TAAs) and one immune cell activation receptor. Examples of such receptors include CD19/CD3/CD20, CD33/CD3/CD123, CD123/NKp46/CD16a, and CD16/IL-15/CD33 (TriKE) [88]. Through this multivalent binding, TsAbs direct immune cells to kill AML cells. In comparison with BsAbs, TsAbs have been shown to reduce escape due to a single target. The incorporation of a third domain, such as IL-15, into TsAbs has been demonstrated to prolong the half-life, facilitate costimulatory signals, and enhance stability [69, 70, 89], thereby achieving dual stimulation signals. Furthermore, TsAbs, through a “weak CD3 and strong tumor bivalent” design, ensure that immune activation occurs only in high antigen density microregions, thereby reducing the risk of CRS.

Arvindam et al. constructed CLL-1-TriKE [85], which uses a CD16a-IL-15-CLEC12A trivalent structure to simultaneously anchor natural killer (NK) cells and AML cells. The IL-15 domain has been demonstrated to amplify the signal autonomously, thereby enabling strong lysis to be triggered at low concentrations. This molecule has been shown to clear over 90% of primary AML and LSC in vitro at concentrations ranging from 0.5 to 5 ng/mL, with significant upregulation of NK degranulation and IFN-γ secretion. In vivo, a dose of 0.1 mg/kg reduced the human CD45 + burden in the bone marrow of NSG mice by more than two logs, with LSC nearly at the detection limit, and no grade > 2 toxicity throughout the process. This finding serves to substantiate the hypothesis that the substance is characterised by elevated efficiency and minimal toxicity, thereby establishing a preclinical foundation for its subsequent clinical translation. Despite the fact that TsAbs are still in the early exploration stage, their innovative design concept has opened up a new treatment pathway for patients with relapsed/refractory (R/R) AML, especially for those who have failed BsAb or CAR-T therapies. By meticulously selecting tumor target antigens, optimising the Fc segment structure, and precisely regulating the intensity of immune activation, TsAbs are anticipated to attain an ideal equilibrium between efficacy and safety in the future, thereby providing a more efficient and tolerable immunotherapy option for clinical utilisation.

### CAR-T cells anti-CLL-1

Chimeric Antigen Receptor T-cell (CAR-T) therapy is a state-of-the-art immunotherapy technology that involves the genetic engineering of T cells to express specific antigen receptors, thereby enabling them to precisely identify and destroy tumor cells [90, 91]. CAR-T therapy has achieved considerable success in the treatment of B-cell malignancies and plasma cell tumors [92, 93], however, its application in AML has been limited by the paucity of suitable targets. CLL-1 is expressed at high levels on over 90% of AML cells, but is either not expressed or expressed at very low levels on normal hematopoietic stem cells. This makes it an ideal antigen target following CD33 and CD123 [94, 95].

Jinghua Wang et al. [96]constructed a second-generation CLL-1 CAR-T that can efficiently and selectively lyse AML blasts and leukemia stem cells in vitro, with no toxicity to normal CD34 + CD38- haematopoietic stem cells. A single dose of 1 × 10⁶ CAR-T cells reduced the leukemia burden in the bone marrow of NSG mice by more than two logs, with no deaths related to CRS or weight loss. This preclinical data established a robust foundation of high efficiency and safety for the clinical translation of CLL-1-targeted CAR-T therapy for relapsed/refractory (R/R) AML. Jin X et al. [33]were the first to evaluate the feasibility of CLL-1-targeted CAR-T cell therapy for R/R AML in humans. This Phase I clinical trial (NCT04388944) enrolled 10 adult patients who had failed multiple lines of therapy. The results demonstrated that following fludarabine-cyclophosphamide lymphodepletion, the infusion of 1–2 × 10⁶ cells/kg of CAR-T cells resulted in an objective response rate of 70%, with three patients attaining minimal residual disease (MRD)-negative complete remission. Furthermore, CLL-1⁺ cells in the bone marrow were undetectable within 28 days, and CAR-T persistence was > 6 months. With regard to safety, all patients experienced CRS, with six cases being high-grade, but no CRES was observed. The principal dose-limiting toxicity was identified as prolonged neutropenia. This study was the first to confirm in humans that CLL-1 CAR-T therapy can induce deep and durable clinical remission, with controllable safety but a need for further optimization of myelosuppression. This study thus lays an important foundation for subsequent clinical development. It is particularly noteworthy that for patients with AML accompanied by extramedullary diseases (EMDs), CLL-1 CAR-T therapy also demonstrated satisfactory efficacy. A clinical investigation (ChiCTR2000041054) [97], revealed that, in a cohort of 20 AML patients with EMDs, the complete remission rate of CLL-1 CAR-T therapy was 65.00%, and that these patients subsequently achieved long-term survival through the process of haematopoietic stem cell transplantation. Current CAR-T treatments targeting CLL-1 are steadily advancing, as evidenced in Table 3, and may provide a new therapeutic strategy to improve the clinical outcomes of patients with R/R AML.

Notwithstanding the encouraging outlook for CLL−1 CAR-T therapy in the treatment of AML, there are numerous challenges to be surmounted before its clinical translation can be realised. In a phase I trial of a chimeric antigen receptor (CAR) T-cell therapy for paediatric relapsed/refractory (R/R) acute lymphoblastic leukaemia (ALL), three patients achieved short-term complete remission. However, the third patient relapsed molecularly on day 30. The results of the flow cytometry analysis indicated that the newly identified leukemia clusters predominantly exhibited a negative expression of the CLL−1 antigen. This finding indicates that while CLL−1 CAR-T therapy can indeed induce remission, its efficacy is transient, and antigen loss emerges as a pivotal mechanism through which leukemia stem cells (LSCs) evade therapy [59]. In order to overcome the phenomenon of antigen escape, the use of multi-target combined CAR-T is indicated. For instance, CD33-CLL−1 CAR-T and CD123-CLL−1 CAR-T utilise a tandem CAR configuration to target two distinct antigens. A study was conducted that targeted CD33 and CLL−1 [35], The study revealed that CD33-CLL−1 CAR-T exhibited a high level of efficacy in eradicating low or negative AML cells (e.g., MOLM−13) in vitro. In a mouse model, CD33-CLL−1 CAR-T significantly reduced tumor load and extended mouse survival. In human experimentation, Liu F’s team employed a P2A self-cleaving peptide to construct a dual-target cCAR. In this study, the patient was a child suffering from relapsed R/R AML (73% PB, 81% BM blasts). The conditioning regimen consisted of fludarabine and cyclophosphamide (Flu/Cy), followed by a two-day infusion of 1 × 10⁶ cCAR-T/kg. On the twelfth day, there was a temporary rise in bone marrow blasts to 98%, but there was significant expansion of CAR-T cells. By the nineteenth day, the patient had achieved MRD-negative CR with 36% PB and 60% BM CAR-T. Thereafter, the child underwent non-myeloablative allo-HSCT, which resulted in a significant reduction in toxicity. This case demonstrates the potential of CD33-CLL−1 cCAR to effectively overcome multidrug resistance, achieving deep remission and establishing a safe transplant window. In addition to dual antigen targets, the combination of CLL−1 CAR-T with epigenetic regulators such as decitabine and panobinostat has been shown to reverse CLL−1 expression in certain AML cells, thereby restoring CAR-T recognition [98].

Toxicity associated with CLL−1 CAR-T therapy is also one of the core challenges in clinical translation. In addition to common CRS and ICANS, myelosuppression and infection are prevalent adverse reactions [99, 100]. Consequently, the potential impact of CLL−1 CAR-T on CLL−1-positive normal cells must be closely monitored and meticulously managed, despite the relatively limited expression of CLL−1 in normal tissues.

**Table 3:** Clinical Trials of Anti-CLL−1 CAR-T Therapy in Acute Myeloid Leukemia

NCT/ChiCTR Number	Simplified Therapeutic Approach (Modified)	Target (Modified)	Population (Modified)	Phase & Status	Key Translational Features
ACTIVE/RECRUITING STUDIES					
NCT07201727	CD123 + CLL-1 Sequential CAR-T → Allo-HSCT bridge	CD123 + CLL-1	R/R AML	Early Phase 1 Not yet recruiting	Sequential dual-targeting + stem cell transplant bridge; curative strategy
NCT07198867	A-CAR028 (CLL-1 CAR-T)	CLL-1	R/R AML	Phase 1 Recruiting	Novel single-target construct
NCT07036250	U32 CAR-T	CLL-1	AML	Phase 1/2 Recruiting	-
NCT06917105	CD33/CD123/CLL-1 Triple CAR-T	CD33 + CD123+CLL-1	Myeloid Malignancies	Phase 1/2 Not yet recruiting	Triple-targeting to address clonal heterogeneity
NCT06880354	CLL-1/CD38 Dual CAR-T	CLL-1 + CD38	AML	Phase 1 Recruiting	Dual-targeting; CD38 adds tumor coverage
NCT06680752	ARD103 CAR-T + Flu/Cy	CLL-1	R/R AML, R/R MDS	Phase 1/2 Recruiting	Extends to MDS; combination with chemotherapy
NCT06347458	BG1805	CLL-1	R/R AML (Pediatric)	Phase 1 Not yet recruiting	Pediatric focus
ChiCTR2500100811	CLL-1 CAR-T	CLL-1	AML	Retrospective Recruiting	Retrospective analysis of existing data
NCT06118788	BG1805	CLL-1	R/R AML	Phase 1/2 Recruiting	Adult population (companion to pediatric NCT06347458)
NCT06027853	CLL-1 CAR-NK	CLL-1	Adult AML	Phase 1 Recruiting	NK cell platform (not T-cell); off-the-shelf potential; reduced CRS risk
NCT06017258	CD371-YSNVz/I-18 CAR-T	CD371 (CLL-1)	AML	Phase 1 Recruiting	Novel construct with IL-18 engineering
NCT05995041	Universal CAR-T (Multi-target)	CLL-1/CD33/CD38/CD123	AML	Phase 1 Recruiting	Universal/allogeneic platform; quadruple targeting
NCT04219163	CLL-1 CAR-T	CLL-1	AML	Phase 1 Active, not recruiting	-
TERMINATED/WITHDRAWN STUDIES					
NCT06128044	CB-012 (CRISPR-edited Allogeneic CAR-T)	CLL-1	R/R AML	Phase 1 Terminated	Gene-edited universal cells (discontinued; important negative data)
NCT06110208	CLL-1/CD38 Dual CAR-T	CLL-1 + CD38	AML	Early Phase 1 Terminated	Dual-targeting (terminated)
NCT05943314	CLL-1/CD33 CAR-T	CLL-1 + CD33	AML	Phase 1 Withdrawn	Dual-targeting (withdrawn)
NCT05654779	LCAR-AMDR (CLL-1/CD33)	CLL-1 + CD33	R/R AML	Phase 1 Terminated	Dual-targeting with AMDR modification (discontinued)
NCT05248685	Dual CD33/CLL-1 CAR-T	CD33 + CLL-1	R/R AML	Phase 1 Withdrawn	Dual-targeting (withdrawn)
UNKNOWN STATUS STUDIES					
ChiCTR1900028303	CLL-1 CAR-T	CLL-1	AML	Phase 1 Unknown	-
ChiCTR2000036350	Multi-target CAR-T (incl. CLL-1)	CD19/20/22/33/123/CLL-1/138/BCMA/CD30	AML	Phase 1 Unknown	Pan-hematologic targeting strategy
NCT05467254	CLL-1 + CD33 CAR-T	CLL-1 + CD33	R/R AML	Phase 1 Unknown	Dual-targeting
NCT05467202	CLL-1 CAR-T	CLL-1	R/R AML	Phase 1 Unknown	-
NCT05252572	CLL-1 CAR-T	CLL-1	AML	Early Phase 1 Unknown	-
NCT05016063	Dual CD33-CLL-1 CAR-T + Flu/Cy	CD33 + CLL-1	R/R AML	Early Phase 1 Unknown	Dual-targeting with lymphodepletion
NCT04923919	Anti-CLL-1 CAR-T	CLL-1	R/R AML	Early Phase 1 Unknown	-
NCT04884984	Anti-CLL-1 CAR-T	CLL-1	R/R AML	Phase 1/2 Unknown	-
NCT04010877	Multi-CAR-T (CLL-1/CD33/CD123)	CLL-1 + CD33+CD123	AML	Phase 1/2 Unknown	Triple-targeting
NCT03795779	CLL-1-CD33 cCAR	CLL-1 + CD33	R/R AML, MDS, MPN, CML	Early Phase 1 Unknown	Dual-targeting; broad myeloid indication
ADVANCED PHASE STUDIES					
NCT03631576	CD123/CLL-1 CAR-T (STPHI_0001)	CD123 + CLL-1	R/R AML	Phase 2/3 Unknown	Most advanced; dual-targeting in late-stage development
NCT03222674	Multi-CAR-T (Muc1/CLL-1/CD123 etc.)	Multiple	AML	Phase 1/2 Unknown	Multi-antigen approach

*AML* Acute Myeloid Leukemia, *MDS* Myelodysplastic Syndrome, *R/R* Relapse/Refractory, *MPN* Myeloproliferative Neoplasm, *CML* Chronic Myeloid Leukemia

### CLL-1 CAR-NK

CAR-NK cells are a type of natural killer (NK) cell that has been genetically modified. The core feature of these cells is the presence of a CAR on their surface, which has the capacity to bind specifically to certain antigens on the AML cell membrane with a high degree of specificity [101, 102]. The fundamental structure encompasses three primary domains: the extracellular antigen recognition domain with a single-chain variable fragment (scFv) targeting the antigen, the transmembrane domain, and the intracellular signaling domain [103, 104]. The CLL-1 CAR-NK cells have been shown to specifically target CLL-1-positive AML cells. Upon contact, the scFv of the CLL-1 antigen on the CAR induces the formation of an immunological synapse. The intracellular signalling modules, such as CD3ζ and 4-1BB/CD28, in the CAR-NK cells rapidly cluster [105], thereby triggering a robust activation cascade. This cascade prompts the NK cells to swiftly transition from a quiescent state to an effector state, thereby acquiring high-efficiency killing capabilities and exerting anti-tumor effects.

Preclinical studies of CLL-1 CAR-NK have achieved key breakthroughs. Pioneering work by Gurney et al. first established CLL-1 CAR in primary NK cells using a non-viral transposon system, while simultaneously knocking out the CISH gene regulating the IL-15 signaling pathway via CRISPR/Cas9 technology [106]. This modification not only significantly enhanced the in vitro expansion capacity and cytotoxic efficacy of CAR-NK cells against AML cell lines, patient primary cells, and LSCs, but also extended median survival from 29 days to over 90 days in primary AML patient-derived xenograft (PDX) mouse models. Some mice achieved potential cure, providing robust evidence for clinical application.

Recently, further engineering strategies have focused on overcoming the bottleneck of insufficient in vivo persistence in traditional CAR-NK cells. These “armored” designs primarily include: (1) co-expressing IL-15 (membrane-bound or secreted) to autonomously provide survival signals, maintaining cell viability and a memory-like phenotype; (2) Dual CAR designs (e.g., simultaneously targeting CLL-1 and CD33) to counteract antigenic heterogeneity in AML and reduce the risk of antigen escape. The 2024 American Society of Hematology (ASH) Annual Meeting reported that CLL-1 CAR-NK cells fortified with “structural optimization + membrane-bound IL-2” demonstrated enhanced in vivo leukemia clearance and prolonged survival benefits in preclinical models. The corresponding first-in-human trial is now in preparation. Additionally, induced pluripotent stem cell (iPSC)-derived CAR-NK platforms (e.g., FT596) offer the potential for standardized, scalable “off-the-shelf” products due to their unlimited expandability and uniform quality, representing a significant frontier in CLL-1 targeted therapy [107].

Compared to CAR-T, CLL-1 CAR-NK possesses multiple potential advantages. CAR-NK cells do not rely on HLA recognition, and allogeneic infusion rarely induces graft-versus-host disease(GVHD) [108]. Their activated cytokine profile primarily consists of IFN-γ and GM-CSF, with minimal production of IL-6 and other cytokines associated with severe CRS. Consequently, the risk of ≥Grade 3 CRS and immune effector cell-associated neurotoxicity syndrome (ICANS) is significantly reduced [109]. Furthermore, while retaining CAR-specific killing, CAR-NK cells preserve innate NK cell functions (e.g., recognition via receptors like NKG2D and DNAM-1, and ADCC), enabling clearance of tumor cells with downregulated target antigens and reducing relapse risk. Benefiting from their inherent allogeneic safety profile, CAR-NK products can be manufactured in advance and cryopreserved, enabling immediate patient access. This addresses core challenges of autologous cell therapies, including lengthy cycles, high costs, and suboptimal cell quality in some patients.

Currently, multiple preclinical and early-stage clinical studies are actively advancing the use of CLL-1 CAR-NK therapy for R/R AML (Table 4). The primary objectives are to validate safety and achieve long-lasting efficacy in vivo, thereby providing AML patients with a novel, highly effective, safe, and readily accessible immunotherapy option.

**Table 4:** Clinical Study of anti CLL-1 CAR-NK Cell Therapy for AML

NCT Number	Therapeutic Approach	Population	Phase & Status	Key Translational Features
NCT06367673	iPSC-derived CAR-NK cells (CLL−1 or CD33 targeted)	Adult AML	Phase 1 Recruiting	iPSC platform: scalable manufacturing, true off-the-shelf potential; dual-targeting capability (CLL−1/CD33 switch)
NCT06307054	CLL−1 CAR-NK cells	R/R AML	Phase 1 Recruiting	CAR-NK platform: inherent safety profile (reduced CRS/ICANS risk vs. CAR-T); no requirement for patient-specific manufacturing
NCT06027853	CLL−1 CAR-NK (cell injection)	Adult AML	Phase 1 Recruiting	Direct CAR-NK administration; standard NK cell engineering approach
NCT05215015	CD33/CLL−1 Dual CAR-NK	AML	Early Phase 1 Unknown	Dual-targeting strategy: simultaneous CD33 + CLL−1 targeting to prevent antigen escape and relapse

*R/R AML* Relapsed/Refractory Acute Myeloid Leukemia, *CRS* Cytokine Release Syndrome, *ICANS* Immune Effector Cell-Associated Neurotoxicity Syndrome, *CAR-NK* Chimeric Antigen Receptor-Natural Killer

## Neutropenia and management

Targeted CLL-1 therapy induces granulocytopenia due to the “targeted/tumor-depletion” effect of targeted treatment on normal myeloid progenitor cells and mature neutrophils, clinically manifesting as widespread and severe pancytopenia. Particularly in CAR-T therapy, the persistent in vivo survival and function of CAR-T cells continuously eliminate newly generated CLL-1-expressing myeloid cells. This leads to long-term, treatment-dependent neutropenia and may even trigger life-threatening infections. Neutropenia is a core risk factor for infection in cancer patients [110]. Following CAR-T therapy targeting CLL-1, nearly all patients experience severe pancytopenia, placing them at extremely high risk of infection [32, 33]. On one hand, CAR-T cells directly recognize and eliminate circulating and bone marrow-resident neutrophils and their progenitor cells expressing CLL-1. On the other hand, intense CRS accompanied by high levels of inflammatory cytokines (such as IL-6 and IFN-γ) may further suppress the bone marrow hematopoietic microenvironment, impairing recovery of all blood cell lines and prolonging the duration of cytopenia [111].

Management strategies for neutropenia should encompass a multidimensional approach covering “monitoring-prevention-intervention.” Supportive care forms the foundation for managing prolonged neutropenia, aiming to prevent and address complications. For high-risk patients with pre-existing neutropenia, prophylactic antimicrobial therapy should be considered. This includes prophylaxis against bacteria, fungi, and viruses. For patients developing granulopenia, consider granulocyte colony-stimulating factor (G-CSF) to promote neutrophil recovery. However, G-CSF use in the CAR-T therapy context requires caution, balancing its benefits in promoting myeloid recovery against the potential risk of exacerbating CRS.

Since residual CAR-T cells continuously eliminate newly emerging CLL-1-positive cells, G-CSF support alone is unlikely to achieve sustained hematopoietic recovery. Therefore, employing CLL-1 CAR-T therapy as a “bridge” strategy following deep clearance of leukemic cells to facilitate allogeneic hematopoietic stem cell transplantation (allo-HSCT) has emerged as the most effective clinical management pathway: transplanting donor hematopoietic systems that do not express this CAR resolves long-term granulocytopenia while leveraging graft-versus-leukemia effects to consolidate efficacy and prevent relapse [32, 33, 112, 113]. Studies have confirmed that bridging allo-HSCT after CLL-1 CAR-T-induced remission successfully achieves complete donor cell chimerism and hematopoietic reconstitution, thereby facilitating long-term survival for patients.

## Immunosuppressive tumor microenvironment

Beyond antigen escape, systemic immune suppression mediated by the TME represents a core challenge limiting the efficacy of CLL-1 targeted therapies. The AML bone marrow microenvironment exhibits highly immunosuppressive characteristics, manifested by enrichment of immunosuppressive cell populations such as regulatory T cells (Tregs), tumor-associated macrophages (TAMs), and myeloid-derived suppressor cells (MDSCs), along with high concentrations of immunosuppressive cytokines like IL-10 and TGF-β. The microenvironment exhibits a state of hypoxia, nutrient deprivation, and elevated metabolic waste (lactate, adenosine) [114]. It drives T-cell exhaustion by downregulating pro-inflammatory cytokine secretion (e.g., IFN-γ) and upregulates immune checkpoint molecules (PD-1, LAG-3, TIM-3), thereby suppressing antitumor activity of T cells and NK cells [115, 116].

Current therapeutic strategies targeting CLL-1 generally lack intrinsic mechanisms to counteract this immunosuppressive microenvironment. Although CLL-1-TriKE effectively induces NK cell activation and IFN-γ production, its mechanism is limited to direct targeting and killing, unable to neutralize inhibitory signals like TGF-β or IL-10 within the microenvironment. Similarly, CLL-1 CAR-T/NK cells are susceptible to metabolic stressors like hypoxia, nutrient deprivation, and lactate accumulation, leading to impaired cell function and insufficient persistence. This vulnerability to microenvironmental suppression underscores the necessity of combination therapy strategies for achieving durable remission. Currently, overcoming TME immunosuppression can be approached through multiple dimensions: (1) Constructing “armored” CAR structures enables effector cells to constitutively secrete pro-inflammatory cytokines like IL-12, IL-15, and IL-18. This suppresses Treg activity and enhances T/NK cell cytotoxicity, effectively countering IL-10/TGF-β-mediated immunosuppression. Additionally, CRISPR/Cas9 gene editing to knockout inhibitory receptors like TGF-β receptor II (TGF-βRII) or NKG2A confers direct resistance to TGF-β in CAR-T/NK cells, significantly enhancing their survival and function within suppressive microenvironments. (2) Combination with immune checkpoint inhibitors: The PD-1/PD-L1 pathway represents a critical immune evasion mechanism in AML, with PD-L1 expression on leukemic blasts strongly correlated with poor prognosis [117]. Combining CLL-1-targeted therapies with PD-1/PD-L1 inhibitors (e.g., nivolumab, pembrolizumab) synergistically enhances antitumor immune responses by reversing T-cell exhaustion and restoring cytotoxic function. Clinical studies demonstrate that combining hypomethylating agents (HMAs, e.g., azacitidine) with PD-1 blockade in R/R AML yields significantly improved response rates [118], suggesting synergistic potential when this combination strategy is used alongside CLL-1-targeted therapies. (3) Targeting metabolic reprogramming to reshape the TME: Modulating glucose metabolism, amino acid metabolism (e.g., arginine, tryptophan), and lipid metabolism within the microenvironment reduces the production of immunosuppressive metabolites like lactate and adenosine, thereby alleviating TME-mediated immune suppression [119–121]. For example, targeting lactate dehydrogenase-A (LDH-A) to lower lactate concentrations helps reverse the impairment of effector cell function caused by low pH environments [122], thereby enhancing the efficacy of anti-CLL-1 therapy.

In summary, the AML bone marrow microenvironment establishes profound immunosuppression through cellular, soluble factor, and metabolic mechanisms, severely limiting the durable efficacy of CLL-1 targeted therapies. Future clinical development should prioritize combination regimens integrating these multidimensional strategies. By enhancing effector cell adaptability, releasing checkpoint inhibition, and reshaping metabolism, comprehensive microenvironment normalization and long-term disease control can be achieved.

## Conclusion

CLL-1 has emerged as a crucial target for precision therapy in AML due to its highly specific expression in LSC and near absence in normal hematopoietic stem cells. The transmembrane receptor structure of the subject is notable for its propensity for rapid internalisation, a property that renders it suitable not only for antibody-mediated cytotoxic reactions, but also for the efficient delivery of drugs or toxins. In recent years, a variety of strategies, including monoclonal antibodies, ADC, BsAbs and trispecific antibodies, and emerging CAR-T and CAR-NK cell therapies, have explored CLL-1 as a target. The findings from preclinical and early clinical trials have provided substantial evidence to support the hypothesis that the substance in question possesses both high specificity and anti-leukaemia activity. Nevertheless, ongoing development must address challenges such as immune escape, heterogeneous expression, an immunosuppressive bone marrow microenvironment, myelosuppression, and CRS/ICANs. It is recommended that future research endeavours concentrate on the synergistic design of other targets, including CD33 and CD123. The employment of dual or trispecific constructs, in conjunction with domain optimisation, has been demonstrated to enhance the sustained killing power of the system. The combination of targeted therapies directed at the CLL-1 antigen with IL-15, or other immune-modulating factors, has the potential to enhance tolerability. The integration of multiple platforms and the adoption of personalised strategies have been identified as key factors in the development of targeted treatments for patients with R/R AML. These treatments have the potential to offer safer, more precise, and sustainable options, representing a significant advancement in the field of cancer treatment.

## Data Availability

No datasets were generated or analyzed during the current study.

## References

[1] Kantarjian H, Kadia T, DiNardo C et al (2021) Acute myeloid leukemia: current progress and future directions[J]. Blood Cancer J 11(2):41. 10.1038/s41408-021-00425-3 33619261 10.1038/s41408-021-00425-3 PMC7900255

[2] Wu Q, Zhong L, Zhang G et al (2025) Complementing therapeutic strategies for acute myeloid leukemia: Signaling pathways and targets of traditional Chinese medicine[J]. Leuk Res 151:107672. 10.1016/j.leukres.2025.107672 40022774 10.1016/j.leukres.2025.107672

[3] Arwanih EY, Louisa M, Rinaldi I et al (2022) Resistance Mechanism of Acute Myeloid Leukemia Cells Against Daunorubicin and Cytarabine. Literature Review[J] Cureus 14(12):e33165. 10.7759/cureus.33165 36726936 10.7759/cureus.33165 PMC9885730

[4] Baena JC, Rosales MC, Estacio M et al (2023) Haploidentical and Matched Sibling Transplantation for Acute Myeloid Leukemia: A Hospital-Based Study[J]. J Hematol 12(6):255–267. 10.14740/jh1162 38188474 10.14740/jh1162 PMC10769648

[5] Perl AE, Martinelli G, Cortes JE et al (2019) Gilteritinib or Chemotherapy for Relapsed or Refractory FLT3-Mutated AML[J]. N Engl J Med 381(18):1728–1740. 10.1056/NEJMoa1902688 31665578 10.1056/NEJMoa1902688

[6] DiNardo CD, Stein EM, de Botton S et al (2018) Durable Remissions with Ivosidenib in IDH1-Mutated Relapsed or Refractory AML[J]. N Engl J Med 378(25):2386–2398. 10.1056/NEJMoa1716984 29860938 10.1056/NEJMoa1716984

[7] Stein EM, DiNardo CD, Pollyea DA et al (2017) Enasidenib in mutant IDH2 relapsed or refractory acute myeloid leukemia[J]. Blood 130(6):722–731. 10.1182/blood-2017-04-779405 28588020 10.1182/blood-2017-04-779405 PMC5572791

[8] Damiani D, Tiribelli M (2025) Monoclonal Antibodies Against Myeloid Leukemia Cells: Current Knowledge and Future Directions[J]. Int J Mol Sci 26(10). 10.3390/ijms26104571 10.3390/ijms26104571 PMC12111345 40429716

[9] Uy GL, Aldoss I, Foster MC et al (2021) Flotetuzumab as salvage immunotherapy for refractory acute myeloid leukemia[J]. Blood 137(6):751–762. 10.1182/blood.2020007732 32929488 10.1182/blood.2020007732 PMC7885824

[10] Pollyea D, Kerre T, Deeren D et al (2025) Downregulation of MICA/MICB improves cell persistence and clinical activity of NKG2DL CAR T-cells in patients with relapsed or refractory acute myeloid leukemia or myelodysplastic neoplasia[J]. Leukemia. 10.1038/s41375-025-02767-4 40954213 10.1038/s41375-025-02767-4

[11] Soleimani SH, Zehtabcheh S, Seraji HR et al (2025) Unveiling the potential of CLL-1: a promising target for AML therapy[J]. Biomark Res 13(1):28. 10.1186/s40364-025-00738-6 39940055 10.1186/s40364-025-00738-6 PMC11823018

[12] van Rhenen A, van Dongen GA, Kelder A et al (2007) The novel AML stem cell associated antigen CLL-1 aids in discrimination between normal and leukemic stem cells[J]. Blood 110(7):2659–2666. 10.1182/blood-2007-03-083048 17609428 10.1182/blood-2007-03-083048

[13] Bill M, Aggerholm A, Kjeldsen E et al (2019) Revisiting CLEC12A as leukaemic stem cell marker in AML: highlighting the necessity of precision diagnostics in patients eligible for targeted therapy[J]. Br J Haematol 184(5):769–781. 10.1111/bjh.15711 30520015 10.1111/bjh.15711

[14] Weis WI, Taylor ME, Drickamer K (1998) The C-type lectin superfamily in the immune system[J]. Immunol Rev 163:19–34. 10.1111/j.1600-065x.1998.tb01185.x 9700499 10.1111/j.1600-065x.1998.tb01185.x

[15] Scur M, Parsons BD, Dey S et al (2023) The diverse roles of C-type lectin-like receptors in immunity[J]. Front Immunol 14:1126043. 10.3389/fimmu.2023.1126043 36923398 10.3389/fimmu.2023.1126043 PMC10008955

[16] Szczykutowicz J, Zimmer M, Orczyk-Pawilowicz M (2025) Amniotic fluid glycoproteins as potential ligands for macrophage galactose-type C-type lectin and their possible implications for immunoregulation during pregnancy[J]. Sci Rep 15(1):32966. 10.1038/s41598-025-16909-2 41006428 10.1038/s41598-025-16909-2 PMC12475167

[17] Tang H, Xiao Y, Qian L et al (2024) Mechanistic insights into the C-type lectin receptor CLEC12A-mediated immune recognition of monosodium urate crystal[J]. J Biol Chem 300(3):105765. 10.1016/j.jbc.2024.105765 38367667 10.1016/j.jbc.2024.105765 PMC10959670

[18] Bakker AB, van den Oudenrijn S, Bakker AQ et al (2004) C-type lectin-like molecule-1: a novel myeloid cell surface marker associated with acute myeloid leukemia[J]. Cancer Res 64(22):8443–8450. 10.1158/0008-5472.CAN-04-1659 15548716 10.1158/0008-5472.CAN-04-1659

[19] Neumann K, Castineiras-Vilarino M, Hockendorf U et al (2014) Clec12a is an inhibitory receptor for uric acid crystals that regulates inflammation in response to cell death[J]. Immunity 40(3):389–399. 10.1016/j.immuni.2013.12.015 24631154 10.1016/j.immuni.2013.12.015

[20] Marshall AS, Willment JA, Lin HH et al (2004) Identification and characterization of a novel human myeloid inhibitory C-type lectin-like receptor (MICL) that is predominantly expressed on granulocytes and monocytes[J]. J Biol Chem 279(15):14792–14802. 10.1074/jbc.M313127200 14739280 10.1074/jbc.M313127200

[21] Bras AE, de Haas V, van Stigt A et al (2019) CD123 expression levels in 846 acute leukemia patients based on standardized immunophenotyping[J]. Cytometry Part B: Clin Cytometry 96(2):134–142. 10.1002/cyto.b.21745 30450744 10.1002/cyto.b.21745 PMC6587863

[22] Aldoss I, Clark M, Song JY et al (2020) Targeting the alpha subunit of IL-3 receptor (CD123) in patients with acute leukemia[J]. Hum vaccines immunotherapeutics 16(10):2341–2348. 10.1080/21645515.2020.1788299 10.1080/21645515.2020.1788299 PMC7644204 32692611

[23] Molica M, Perrone S, Mazzone C et al (2021) CD33 Expression and Gentuzumab Ozogamicin in Acute Myeloid Leukemia: Two Sides of the Same Coin[J]. Cancers 13(13):3214. 10.3390/cancers13133214 34203180 10.3390/cancers13133214 PMC8268215

[24] Majeti R, Chao MP, Alizadeh AA et al (2009) CD47 Is an Adverse Prognostic Factor and Therapeutic Antibody Target on Human Acute Myeloid Leukemia Stem Cells[J]. Cell 138(2):286–299. 10.1016/j.cell.2009.05.045 19632179 10.1016/j.cell.2009.05.045 PMC2726837

[25] Melo Garcia L, Barabé F (2021) Harnessing Macrophages through the Blockage of CD47: Implications for Acute Myeloid Leukemia[J]. Cancers 13(24):6258. 10.3390/cancers13246258 34944878 10.3390/cancers13246258 PMC8699809

[26] Xu L, Xu J, Ma S et al (2017) High Tim-3 expression on AML blasts could enhance chemotherapy sensitivity[J]. Oncotarget 8(60):102088–102096. 10.18632/oncotarget.22141 29254227 10.18632/oncotarget.22141 PMC5731937

[27] Kikushige Y, Shima T, Takayanagi S et al (2010) TIM-3 Is a Promising Target to Selectively Kill Acute Myeloid Leukemia Stem Cells[J]. Cell Stem Cell 7(6):708–717. 10.1016/j.stem.2010.11.014 21112565 10.1016/j.stem.2010.11.014

[28] Wang J, Li H, Kulkarni A et al (2025) Differential impact of TIM-3 ligands on NK cell function[J]. J Immunother Cancer 13(1):e10618. 10.1136/jitc-2024-010618 10.1136/jitc-2024-010618 PMC11748930 39773563

[29] Ma H, Padmanabhan IS, Parmar S et al (2019) Targeting CLL-1 for acute myeloid leukemia therapy[J]. J Hematol Oncol 12(1):41. 10.1186/s13045-019-0726-5 31014360 10.1186/s13045-019-0726-5 PMC6480870

[30] Wang J, Wang W, Chen H et al (2021) C-Type Lectin-Like Molecule-1 as a Biomarker for Diagnosis and Prognosis in Acute Myeloid Leukemia: A Preliminary Study[J]. Biomed Res Int 2021:6643948. 10.1155/2021/6643948 10.1155/2021/6643948 PMC7979301 33778076

[31] Wang YY, Chen WL, Weng XQ et al (2017) Low CLL-1 Expression Is a Novel Adverse Predictor in 123 Patients with De Novo CD34(+) Acute Myeloid Leukemia[J]. Stem Cells Dev 26(20):1460–1467. 10.1089/scd.2016.0310 28810819 10.1089/scd.2016.0310 PMC5651960

[32] Lloret-Madrid P, Chorão P, Guerreiro M et al (2025) CAR-T Cell Therapy for Acute Myeloid Leukemia: Where Do We Stand Now?[J]. Current oncology (Toronto, Ont.) 32(6):322. 10.3390/curroncol32060322 10.3390/curroncol32060322 PMC12191959 40558265

[33] Jin X, Zhang M, Sun R et al (2022) First-in-human phase I study of CLL-1 CAR-T cells in adults with relapsed/refractory acute myeloid leukemia[J]. J Hematol Oncol 15(1):88. 10.1186/s13045-022-01308-1 35799191 10.1186/s13045-022-01308-1 PMC9264641

[34] van Loo PF, Hangalapura BN, Thordardottir S et al (2019) MCLA-117, a CLEC12AxCD3 bispecific antibody targeting a leukaemic stem cell antigen, induces T cell-mediated AML blast lysis[J]. Expert Opin Biol Ther 19(7):721–733. 10.1080/14712598.2019.1623200 31286786 10.1080/14712598.2019.1623200

[35] Wang H, Feng S, Zhu Y et al (2025) The tandem CD33-CLL1 CAR-T as an approach to treat acute myeloid leukemia[J]. Blood Transfus 23(4):338–347. 10.2450/BloodTransfus.786 39133622 10.2450/BloodTransfus.786 PMC12274197

[36] Wang X, Bian M, Lin G et al (2024) Tandem bispecific CD123/CLL-1 CAR-T cells exhibit specific cytolytic effector functions against human acute myeloid leukaemia[J]. Eur J Haematol 112(1):83–93. 10.1111/ejh.14104 37712633 10.1111/ejh.14104

[37] Malamud M, Brown GD (2024) The Dectin-1 and Dectin-2 clusters: C-type lectin receptors with fundamental roles in immunity[J]. EMBO Rep 25(12):5239–5264. 10.1038/s44319-024-00296-2 39482490 10.1038/s44319-024-00296-2 PMC11624271

[38] Pare G, Vitry J, Merchant ML et al (2021) The Inhibitory Receptor CLEC12A Regulates PI3K-Akt Signaling to Inhibit Neutrophil Activation and Cytokine Release[J]. Front Immunol 12:650808. 10.3389/fimmu.2021.650808 34234773 10.3389/fimmu.2021.650808 PMC8256872

[39] Ben-Khemis M, Liu D, Pintard C et al (2023) TNFalpha counteracts interleukin-10 anti-inflammatory pathway through the NOX2-Lyn-SHP-1 axis in human monocytes[J]. Redox Biol 67:102898. 10.1016/j.redox.2023.102898 37757542 10.1016/j.redox.2023.102898 PMC10539668

[40] Pang J, Cen C, Tian Y et al (2025) Targeting Shp2 as a therapeutic strategy for neurodegenerative diseases[J]. Transl Psychiatry 15(1):6. 10.1038/s41398-024-03222-1 39794316 10.1038/s41398-024-03222-1 PMC11724000

[41] Liu J, Wei Y, Jia W et al (2022) Chenodeoxycholic acid suppresses AML progression through promoting lipid peroxidation via ROS/p38 MAPK/DGAT1 pathway and inhibiting M2 macrophage polarization[J]. Redox Biol 56:102452. 10.1016/j.redox.2022.102452 36084349 10.1016/j.redox.2022.102452 PMC9465103

[42] Chen CH, Floyd H, Olson NE et al (2006) Dendritic-cell-associated C-type lectin 2 (DCAL-2) alters dendritic-cell maturation and cytokine production[J]. Blood 107(4):1459–1467. 10.1182/blood-2005-08-3264 16239426 10.1182/blood-2005-08-3264 PMC1895401

[43] Canton M, Sanchez-Rodriguez R, Spera I et al (2021) Reactive Oxygen Species in Macrophages: Sources and Targets[J]. Front Immunol 12:734229. 10.3389/fimmu.2021.734229 34659222 10.3389/fimmu.2021.734229 PMC8515906

[44] Dilly S, Romero M, Solier S et al (2023) Targeting M2 Macrophages with a Novel NADPH Oxidase Inhibitor[J]. Antioxidants (Basel) 12(2). 10.3390/antiox12020440 10.3390/antiox12020440 PMC9951936 36830003

[45] Zhao X, Singh S, Pardoux C et al (2010) Targeting C-type lectin-like molecule-1 for antibody-mediated immunotherapy in acute myeloid leukemia[J]. Haematologica 95(1):71–78. 10.3324/haematol.2009.009811 19648166 10.3324/haematol.2009.009811 PMC2805740

[46] Jiang YP, Liu BY, Zheng Q et al (2018) CLT030, a leukemic stem cell-targeting CLL1 antibody-drug conjugate for treatment of acute myeloid leukemia[J]. Blood Adv 2(14):1738–1749. 10.1182/bloodadvances.2018020107 30037800 10.1182/bloodadvances.2018020107 PMC6058235

[47] Drent E, Poels R, Ruiter R et al (2019) Combined CD28 and 4-1BB Costimulation Potentiates Affinity-tuned Chimeric Antigen Receptor-engineered T Cells[J]. Clin Cancer Res 25(13):4014–4025. 10.1158/1078-0432.CCR-18-2559 30979735 10.1158/1078-0432.CCR-18-2559 PMC7477921

[48] Zhang X, Cheng X, Yu L et al (2016) MCOLN1 is a ROS sensor in lysosomes that regulates autophagy[J]. Nat Commun 7:12109. 10.1038/ncomms12109 27357649 10.1038/ncomms12109 PMC4931332

[49] Klimovich B, Anton L, Jung J et al (2025) CLEC12A-directed immunocytokine with target cell-restricted IL-15 activity for treatment of acute myeloid leukemia[J]. Front Immunol 16:1561823. 10.3389/fimmu.2025.1561823 40213559 10.3389/fimmu.2025.1561823 PMC11983603

[50] Orange JS (2008) Formation and function of the lytic NK-cell immunological synapse[J]. Nat Rev Immunol 8(9):713–725. 10.1038/nri2381 19172692 10.1038/nri2381 PMC2772177

[51] Daver N, Salhotra A, Brandwein JM et al (2021) A Phase I dose-escalation study of DCLL9718S, an antibody-drug conjugate targeting C-type lectin-like molecule-1 (CLL-1) in patients with acute myeloid leukemia[J]. Am J Hematol 96(5):E175–E179. 10.1002/ajh.26136 33617672 10.1002/ajh.26136 PMC8252033

[52] Tang Z, Xie Y, Zeng Y (2025) Antibody–drug conjugate: a newly developed biological missile for tumor treatment[J]. Front Oncol 15:1688057. 10.3389/fonc.2025.1688057 41234717 10.3389/fonc.2025.1688057 PMC12605357

[53] Zhou Q, Kyazike J, Boudanova E et al (2021) Site-Specific Antibody Conjugation to Engineered Double Cysteine Residues[J]. Pharmaceuticals (Basel) 14(7). 10.3390/ph14070672 10.3390/ph14070672 PMC8308878 34358098

[54] Axup JY, Bajjuri KM, Ritland M et al (2012) Synthesis of site-specific antibody-drug conjugates using unnatural amino acids[J]. Proc Natl Acad Sci U S A 109(40):16101–16106. 10.1073/pnas.1211023109 22988081 10.1073/pnas.1211023109 PMC3479532

[55] Popp MW, Ploegh HL (2011) Making and breaking peptide bonds: protein engineering using sortase[J]. Angew Chem Int Ed Engl 50(22):5024–5032. 10.1002/anie.201008267 21538739 10.1002/anie.201008267

[56] Boswell CA, Mundo EE, Zhang C et al (2011) Impact of drug conjugation on pharmacokinetics and tissue distribution of anti-STEAP1 antibody-drug conjugates in rats[J]. Bioconjug Chem 22(10):1994–2004. 10.1021/bc200212a 21913715 10.1021/bc200212a

[57] Tumey LN, Charati M, He T et al (2014) Mild method for succinimide hydrolysis on ADCs: impact on ADC potency, stability, exposure, and efficacy[J]. Bioconjug Chem 25(10):1871–1880. 10.1021/bc500357n 25216346 10.1021/bc500357n

[58] Beck A, Goetsch L, Dumontet C et al (2017) Strategies and challenges for the next generation of antibody–drug conjugates[J]. Nat Rev Drug Discovery 16(5):315–337. 10.1038/nrd.2016.268 28303026 10.1038/nrd.2016.268

[59] Zhang H, Wang P, Li Z et al (2021) Anti-CLL1 Chimeric Antigen Receptor T-Cell Therapy in Children with Relapsed/Refractory Acute Myeloid Leukemia[J]. Clin Cancer Res 27(13):3549–3555. 10.1158/1078-0432.CCR-20-4543 33832948 10.1158/1078-0432.CCR-20-4543

[60] Qi QR, Tian H, Yue BS et al (2024) Research Progress of SN38 Drug Delivery System in Cancer Treatment[J]. Int J Nanomed 19:945–964. 10.2147/IJN.S435407 10.2147/IJN.S435407 PMC10826519 38293612

[61] Wen M, Yu A, Park Y et al (2025) Homogeneous antibody-drug conjugates with dual payloads: potential, methods and considerations[J]. mAbs 17(1):2498162. 10.1080/19420862.2025.2498162 40322862 10.1080/19420862.2025.2498162 PMC12054377

[62] Madsen AV, Pedersen LE, Kristensen P et al (2024) Design and engineering of bispecific antibodies: insights and practical considerations[J]. Front Bioeng Biotechnol 12:1352014. 10.3389/fbioe.2024.1352014 38333084 10.3389/fbioe.2024.1352014 PMC10850309

[63] Labrijn AF, Janmaat ML, Reichert JM et al (2019) Bispecific antibodies: a mechanistic review of the pipeline[J]. Nat Rev Drug Discov 18(8):585–608. 10.1038/s41573-019-0028-1 31175342 10.1038/s41573-019-0028-1

[64] Sun Y, Yu X, Wang X et al (2023) Bispecific antibodies in cancer therapy: Target selection and regulatory requirements[J]. Acta Pharm Sin B 13(9):3583–3597. 10.1016/j.apsb.2023.05.023 37719370 10.1016/j.apsb.2023.05.023 PMC10501874

[65] Bonnevaux H, Guerif S, Albrecht J et al (2021) Pre-clinical development of a novel CD3-CD123 bispecific T-cell engager using cross-over dual-variable domain (CODV) format for acute myeloid leukemia (AML) treatment[J]. Oncoimmunology 10(1):1945803. 10.1080/2162402X.2021.1945803 34484869 10.1080/2162402X.2021.1945803 PMC8409758

[66] Zhong L, Shi W, Gan L et al (2021) Human endoglin-CD3 bispecific T cell engager antibody induces anti-tumor effect in vivo[J]. Theranostics 11(13):6393–6406. 10.7150/thno.53121 33995664 10.7150/thno.53121 PMC8120215

[67] Takahashi K (2022) [Novel therapies in AML and their resistance mechanisms][J]. Rinsho Ketsueki 63(9):1052–1057. 10.11406/rinketsu.63.1052 36198529 10.11406/rinketsu.63.1052

[68] Kantarjian H, Stein A, Gokbuget N et al (2017) Blinatumomab versus Chemotherapy for Advanced Acute Lymphoblastic Leukemia[J]. N Engl J Med 376(9):836–847. 10.1056/NEJMoa1609783 28249141 10.1056/NEJMoa1609783 PMC5881572

[69] Zhu A, Bai Y, Nan Y et al (2024) Natural killer cell engagers: From bi-specific to tri-specific and tetra-specific engagers for enhanced cancer immunotherapy[J]. Clin Transl Med 14(11):e70046. 10.1002/ctm2.70046 39472273 10.1002/ctm2.70046 PMC11521791

[70] Ramirez-Labrada A, Pesini C, Santiago L et al (2022) All About (NK Cell-Mediated) Death in Two Acts and an Unexpected Encore: Initiation, Execution and Activation of Adaptive Immunity[J]. Front Immunol 13:896228. 10.3389/fimmu.2022.896228 35651603 10.3389/fimmu.2022.896228 PMC9149431

[71] Kontermann RE (2012) Dual targeting strategies with bispecific antibodies[J]. MAbs 4(2):182–197. 10.4161/mabs.4.2.19000 22453100 10.4161/mabs.4.2.19000 PMC3361654

[72] Schanzer JM, Wartha K, Croasdale R et al (2014) A novel glycoengineered bispecific antibody format for targeted inhibition of epidermal growth factor receptor (EGFR) and insulin-like growth factor receptor type I (IGF-1R) demonstrating unique molecular properties[J]. J Biol Chem 289(27):18693–18706. 10.1074/jbc.M113.528109 24841203 10.1074/jbc.M113.528109 PMC4081915

[73] Li Y, Hickson JA, Ambrosi DJ et al (2018) ABT-165, a Dual Variable Domain Immunoglobulin (DVD-Ig) Targeting DLL4 and VEGF, Demonstrates Superior Efficacy and Favorable Safety Profiles in Preclinical Models[J]. Mol Cancer Ther 17(5):1039–1050. 10.1158/1535-7163.MCT-17-0800 29592882 10.1158/1535-7163.MCT-17-0800

[74] Chen RP, Shinoda K, Rampuria P et al (2022) Bispecific antibodies for immune cell retargeting against cancer[J]. Expert Opin Biol Ther 22(8):965–982. 10.1080/14712598.2022.2072209 35485219 10.1080/14712598.2022.2072209

[75] Cheng L, Chen L, Shi Y et al (2024) Efficacy and safety of bispecific antibodies vs. immune checkpoint blockade combination therapy in cancer: a real-world comparison[J]. Mol Cancer 23(1):77. 10.1186/s12943-024-01956-6 38627681 10.1186/s12943-024-01956-6 PMC11020943

[76] Huang PL, Kan HT, Hsu CH et al (2023) A bispecific antibody AP203 targeting PD-L1 and CD137 exerts potent antitumor activity without toxicity[J]. J Transl Med 21(1):346. 10.1186/s12967-023-04193-5 37226226 10.1186/s12967-023-04193-5 PMC10210478

[77] Zhao Y, Chen G, Li X et al (2024) KN046, a bispecific antibody against PD-L1 and CTLA-4, plus chemotherapy as first-line treatment for metastatic NSCLC: A multicenter phase 2 trial[J]. Cell Rep Med 5(3):101470. 10.1016/j.xcrm.2024.101470 38508135 10.1016/j.xcrm.2024.101470 PMC10983105

[78] Zhao Y, Ma Y, Fan Y et al (2023) A multicenter, open-label phase Ib/II study of cadonilimab (anti PD-1 and CTLA-4 bispecific antibody) monotherapy in previously treated advanced non-small-cell lung cancer (AK104-202 study)[J]. Lung Cancer 184:107355. 10.1016/j.lungcan.2023.107355 37677918 10.1016/j.lungcan.2023.107355

[79] Haber L, Olson K, Kelly MP et al (2021) Generation of T-cell-redirecting bispecific antibodies with differentiated profiles of cytokine release and biodistribution by CD3 affinity tuning[J]. Sci Rep 11(1):14397. 10.1038/s41598-021-93842-0 34257348 10.1038/s41598-021-93842-0 PMC8277787

[80] Lee E, Lee S, Park S et al (2023) Asymmetric anti-CLL-1xCD3 bispecific antibody, ABL602 2 + 1, with attenuated CD3 affinity endows potent antitumor activity but limited cytokine release[J]. J Immunother Cancer 11(10). 10.1136/jitc-2023-007494 10.1136/jitc-2023-007494 PMC10582864 37848261

[81] Benard BA, Leak LB, Azizi A et al (2021) Clonal architecture predicts clinical outcomes and drug sensitivity in acute myeloid leukemia[J]. Nat Commun 12(1):7244. 10.1038/s41467-021-27472-5 34903734 10.1038/s41467-021-27472-5 PMC8669028

[82] Lindblad KE, Goswami M, Hourigan CS et al (2017) Immunological effects of hypomethylating agents[J]. Expert Rev Hematol 10(8):745–752. 10.1080/17474086.2017.1346470 28644756 10.1080/17474086.2017.1346470 PMC6071309

[83] Wong KK, Hassan R, Yaacob NS (2021) Hypomethylating Agents and Immunotherapy: Therapeutic Synergism in Acute Myeloid Leukemia and Myelodysplastic Syndromes[J]. Front Oncol 11:624742. 10.3389/fonc.2021.624742 33718188 10.3389/fonc.2021.624742 PMC7947882

[84] Derer S, Kellner C, Berger S et al (2012) Fc engineering: design, expression, and functional characterization of antibody variants with improved effector function[J]. Methods Mol Biol 907:519–536. 10.1007/978-1-61779-974-7_30 22907372 10.1007/978-1-61779-974-7_30

[85] Arvindam US, van Hauten P, Schirm D et al (2021) A trispecific killer engager molecule against CLEC12A effectively induces NK-cell mediated killing of AML cells[J]. Leukemia 35(6):1586–1596. 10.1038/s41375-020-01065-5 33097838 10.1038/s41375-020-01065-5 PMC8189652

[86] Krawczyk E, Zolov SN, Huang K et al (2019) T-cell Activity against AML Improved by Dual-Targeted T Cells Stimulated through T-cell and IL7 Receptors[J]. Cancer Immunol Res 7(4):683–692. 10.1158/2326-6066.CIR-18-0748 30782669 10.1158/2326-6066.CIR-18-0748 PMC8186236

[87] Leong SR, Sukumaran S, Hristopoulos M et al (2017) An anti-CD3/anti-CLL-1 bispecific antibody for the treatment of acute myeloid leukemia[J]. Blood 129(5):609–618. 10.1182/blood-2016-08-735365 27908880 10.1182/blood-2016-08-735365 PMC5290988

[88] Tapia-Galisteo A, Compte M, Alvarez-Vallina L et al (2023) When three is not a crowd: trispecific antibodies for enhanced cancer immunotherapy[J]. Theranostics 13(3):1028–1041. 10.7150/thno.81494 36793863 10.7150/thno.81494 PMC9925307

[89] Felices M, Lenvik TR, Davis ZB et al (2016) Generation of BiKEs and TriKEs to Improve NK Cell-Mediated Targeting of Tumor Cells[J]. Methods Mol Biol 1441:333–346. 10.1007/978-1-4939-3684-7_28 27177679 10.1007/978-1-4939-3684-7_28 PMC5823010

[90] June CH, O’Connor RS, Kawalekar OU et al (2018) CAR T cell immunotherapy for human cancer[J]. Science 359(6382):1361–1365. 10.1126/science.aar6711 29567707 10.1126/science.aar6711

[91] Brentjens RJ, Davila ML, Riviere I et al (2013) CD19-targeted T cells rapidly induce molecular remissions in adults with chemotherapy-refractory acute lymphoblastic leukemia[J]. Sci Transl Med 5(177):177ra38. 10.1126/scitranslmed.3005930 23515080 10.1126/scitranslmed.3005930 PMC3742551

[92] He B, Lin R, Xu N et al (2025) Efficacy and safety of third-generation CD19-CAR T cells incorporating CD28 and TLR2 intracellular domains for B-cell malignancies with central nervous system involvement: results of a pivotal trial[J]. J Transl Med 23(1):594. 10.1186/s12967-025-06608-x 40426201 10.1186/s12967-025-06608-x PMC12117732

[93] Raje N, Berdeja J, Lin Y et al (2019) Anti-BCMA CAR T-Cell Therapy bb2121 in Relapsed or Refractory Multiple Myeloma[J]. N Engl J Med 380(18):1726–1737. 10.1056/NEJMoa1817226 31042825 10.1056/NEJMoa1817226 PMC8202968

[94] Laborda E, Mazagova M, Shao S et al (2017) Development of A Chimeric Antigen Receptor Targeting C-Type Lectin-Like Molecule-1 for Human Acute Myeloid Leukemia[J]. Int J Mol Sci 18(11). 10.3390/ijms18112259 10.3390/ijms18112259 PMC5713229 29077054

[95] Wang X, Zhang Y, Xue S (2024) Recent progress in chimeric antigen receptor therapy for acute myeloid leukemia[J]. Ann Hematol 103(6):1843–1857. 10.1007/s00277-023-05601-y 38381173 10.1007/s00277-023-05601-y

[96] Wang J, Chen S, Xiao W et al (2018) CAR-T cells targeting CLL-1 as an approach to treat acute myeloid leukemia[J]. J Hematol Oncol 11(1):7. 10.1186/s13045-017-0553-5 29316944 10.1186/s13045-017-0553-5 PMC5761206

[97] Zhao Y, Bai X, Guo S et al (2024) Efficacy and safety of CAR-T therapy targeting CLL1 in patients with extramedullary diseases of acute myeloid leukemia[J]. J Transl Med 22(1):888. 10.1186/s12967-024-05705-7 39358720 10.1186/s12967-024-05705-7 PMC11446059

[98] Nan F, Fu X, Chen X et al (2022) Strategies to overcome CAR-T cell resistance in clinical work: A single-institute experience[J]. Front Immunol 13:929221. 10.3389/fimmu.2022.929221 36032118 10.3389/fimmu.2022.929221 PMC9399606

[99] Xu J, Zhang H, Zhao Y et al (2025) Infectious complications distribution following CLL1 CAR-T cell therapy for acute myeloid leukemiass[J]. Cancer Immunol Immunother 74(5):149. 10.1007/s00262-025-03998-1 40088283 10.1007/s00262-025-03998-1 PMC11910464

[100] Han MW, Jeong SY, Suh CH et al (2024) Incidence of immune effector cell-associated neurotoxicity among patients treated with CAR T-cell therapy for hematologic malignancies: systematic review and meta-analysis[J]. Front Neurol 15:1392831. 10.3389/fneur.2024.1392831 39474369 10.3389/fneur.2024.1392831 PMC11518750

[101] Zhang L, Meng Y, Yao H et al (2023) CAR-NK cells for acute myeloid leukemia immunotherapy: past, present and future[J]. Am J Cancer Res 13(11):5559–5576 38058830 PMC10695781

[102] Bashiri DA, Yazdi M, Pockley AG et al (2021) NK Cells Armed with Chimeric Antigen Receptors (CAR): Roadblocks to Successful Development[J]. Cells 10(12). 10.3390/cells10123390 10.3390/cells10123390 PMC8699535 34943898

[103] Chang ZL, Chen YY, CARs (2017) Synthetic Immunoreceptors for Cancer Therapy and Beyond[J]. Trends Mol Med 23(5):430–450. 10.1016/j.molmed.2017.03.002 28416139 10.1016/j.molmed.2017.03.002 PMC5423782

[104] Yi E, Lee E, Park HJ et al (2025) A chimeric antigen receptor tailored to integrate complementary activation signals potentiates the antitumor activity of NK cells[J]. J Exp Clin Cancer Res 44(1):86. 10.1186/s13046-025-03351-5 40045373 10.1186/s13046-025-03351-5 PMC11884141

[105] Grote S, Mittelstaet J, Baden C et al (2020) Adapter chimeric antigen receptor (AdCAR)-engineered NK-92 cells: an off-the-shelf cellular therapeutic for universal tumor targeting[J]. Oncoimmunology 9(1):1825177. 10.1080/2162402X.2020.1825177 33457105 10.1080/2162402X.2020.1825177 PMC7781805

[106] Gurney M, O’Reilly E, Corcoran S et al (2022) Concurrent transposon engineering and CRISPR/Cas9 genome editing of primary CLL-1 chimeric antigen receptor-natural killer cells[J]. Cytotherapy 24(11):1087–1094. 10.1016/j.jcyt.2022.07.008 36050244 10.1016/j.jcyt.2022.07.008

[107] Kong R, Liu B, Wang H et al (2025) CAR-NK cell therapy: latest updates from the 2024 ASH annual meeting[J]. J Hematol Oncol 18(1):22. 10.1186/s13045-025-01677-3 10.1186/s13045-025-01677-3 PMC11872314 40025557

[108] Simonetta F, Alvarez M, Negrin RS (2017) Natural Killer Cells in Graft-versus-Host-Disease after Allogeneic Hematopoietic Cell Transplantation[J]. Front Immunol 8:465. 10.3389/fimmu.2017.00465 10.3389/fimmu.2017.00465 PMC5403889 28487696

[109] Lei W, Liu H, Deng W et al (2025) Safety and feasibility of 4-1BB co-stimulated CD19-specific CAR-NK cell therapy in refractory/relapsed large B cell lymphoma: a phase 1 trial[J]. Nat Cancer 6(5):786–800. 10.1038/s43018-025-00940-3 40251398 10.1038/s43018-025-00940-3 PMC12122374

[110] Yacoub AT, Krishnan J, Acevedo IM et al (2015) Nutritionally variant streptococci bacteremia in cancer patients: a retrospective study, 1999–2014[J]. Mediterranean J Hematol Infect Dis 7(1):e2015030. 10.4084/MJHID.2015.030 10.4084/MJHID.2015.030 PMC4418387 25960858

[111] Goedhart M, Cornelissen AS, Kuijk C et al (2018) nterferon-Gamma Impairs Maintenance and Alters Hematopoietic Support of Bone Marrow Mesenchymal Stromal Cells[J]. Stem Cells Dev 27(9):579–589. 10.1089/scd.2017.0196 10.1089/scd.2017.0196 PMC5934977 29649408

[112] Miao X, Shuai Y, Han Y et al (2024) Case report: Donor-derived CLL-1 chimeric antigen receptor T-cell therapy for relapsed/refractory acute myeloid leukemia bridging to allogeneic hematopoietic stem cell transplantation after remission[J]. Front Immunol 15:1389227. 10.3389/fimmu.2024.1389227 38803489 10.3389/fimmu.2024.1389227 PMC11128603

[113] Kadirkamanathan R, Georgiadis C, Kloos A et al (2025) Base edited universal donor CAR T-cell strategies for acute myeloid leukaemia[J]. Leukemia 39(12):2978–2987. 10.1038/s41375-025-02720-5 41034424 10.1038/s41375-025-02720-5 PMC12634419

[114] Li W, Chen J, Li J et al (2025) Envirotune-CAR-T: a hypoxia-responsive and glutamine-enhanced CAR-T cell therapy for overcoming tumor microenvironment-mediated suppression[J]. J Immunother Cancer 13(10):e12321. 10.1136/jitc-2025-012321 10.1136/jitc-2025-012321 PMC12519721 41083281

[115] Matamala Montoya M, van Slobbe GJJ, Chang J et al (2023) Metabolic changes underlying drug resistance in the multiple myeloma tumor microenvironment[J]. Front Oncol 13:1155621. 10.3389/fonc.2023.1155621 37091139 10.3389/fonc.2023.1155621 PMC10117897

[116] Wu S, Kuang H, Ke J et al (2021) Metabolic Reprogramming Induces Immune Cell Dysfunction in the Tumor Microenvironment of Multiple Myeloma[J]. Front Oncol 10:591342. 10.3389/fonc.2020.591342 33520703 10.3389/fonc.2020.591342 PMC7845572

[117] Brodská B, Otevřelová P, Šálek C et al (2019) High PD-L1 Expression Predicts for Worse Outcome of Leukemia Patients with Concomitant NPM1 and FLT3 Mutations[J]. Int J Mol Sci 20(11):2823. 10.3390/ijms20112823 31185600 10.3390/ijms20112823 PMC6600137

[118] Daver N, Garcia-Manero G, Basu S et al (2019) Efficacy, Safety, and Biomarkers of Response to Azacitidine and Nivolumab in Relapsed/Refractory Acute Myeloid Leukemia: A Nonrandomized, Open-Label, Phase II Study[J]. Cancer Discov 9(3):370–383. 10.1158/2159-8290.CD-18-0774 30409776 10.1158/2159-8290.CD-18-0774 PMC6397669

[119] Chen J, Huang Z, Chen Y et al (2025) Lactate and lactylation in cancer[J]. Signal Transduct Target therapy 10(1):38. 10.1038/s41392-024-02082-x 10.1038/s41392-024-02082-x PMC11814237 39934144

[120] Ma X, Xiao L, Liu L et al (2021) CD36-mediated ferroptosis dampens intratumoral CD8(+) T cell effector function and impairs their antitumor ability[J]. Cell Metabol 33(5):1001–1012. 10.1016/j.cmet.2021.02.015 10.1016/j.cmet.2021.02.015 PMC8102368 33691090

[121] Zhang Y, Feng S, Lv L et al (2025) Amino Acid Metabolism-Related Gene Kynureninase (KYNU) as a Prognostic Predictor and Regulator of Diffuse Large B-Cell Lymphoma[J]. Biochem Genet 10–1007. 10.1007/s10528-025-11047-w 10.1007/s10528-025-11047-w 39932613

[122] Mane MM, Cohen IJ, Ackerstaff E et al (2020) Lactate Dehydrogenase A Depletion Alters MyC-CaP Tumor Metabolism, Microenvironment, and CAR T Cell Therapy[J]. Mol therapy oncolytics 18:382–395. 10.1016/j.omto.2020.07.006 10.1016/j.omto.2020.07.006 PMC7452096 32913888

